# Tunable Structural Color in Copolymer Microgels Through Controlled Synthesis and Thermally Induced Assembly

**DOI:** 10.1002/smsc.202600001

**Published:** 2026-03-30

**Authors:** Manuel Kraus, Mirela Malekovic, Ionel Adrian Dinu, Cornelia G. Palivan

**Affiliations:** ^1^ Department of Chemistry University of Basel Basel Switzerland; ^2^ Swiss Nanoscience Institute University of Basel Basel Switzerland

**Keywords:** colloidal assembly, dynamic structural color, functional comonomers, poly(N‐isopropylacrylamide) (PNIPAm)‐based copolymer microgels, temperature‐ and pH‐responsiveness

## Abstract

Structural color formation in soft colloidal systems represents a promising approach toward stimuli‐responsive photonic materials. In this study, the assembly of poly(N‐isopropylacrylamide) (PNIPAm)‐based microgels incorporating varying percentages of anionic (methacrylic acid), neutral (acrylamide), and cationic (2‐(dimethylamino)ethyl methacrylate) comonomers to create multifunctional colloidal building blocks capable of forming dynamic structural colors was systematically investigated. Through optimization of synthesis conditions, including comonomer selection, their concentration, and surfactant‐mediated size control, libraries of copolymer microgels with precisely tailored particle diameter, surface charge, and stimuli‐responsive swelling behavior were obtained. Structural color tuning across the visible spectrum was generated by thermal colloidal assembly. The interparticle spacing and consequently the reflected wavelengths were modulated by microgel concentration and their stimuli‐responsive behavior: Finely tuned responsiveness of the microgels allowed for precise shifts in the reflected color upon environmental stimuli (temperature or pH). Such changes of the structural colors upon stimuli were clearly observable in the colloidal crystal assemblies of anionic and neutral microgels, while the amorphous assembly of the cationic microgels limited the detection of small shifts. Our findings provide key insights into the interplay between microgel responsiveness, compressibility, and the resulting structural color formation that are expected to support advanced multifunctional colloidal sensors, coatings, and optical tags.

## Graphical abstract

Poly(N‐isopropylacrylamide) (PNIPAm)‐based copolymer microgels with incorporated anionic, cationic, and neutral comonomers served as multifunctional colloidal building blocks for the formation of dynamic structural colors. Precise control over synthesis parameters and self‐assembly behavior allow for the fine‐tuning of the reflected color across the entire visible spectrum and sensing of environmental stimuli such as temperature and pH.

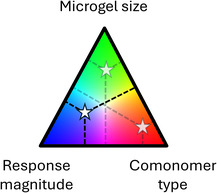

## 1 Introduction

Structural colors represent an attractive alternative to conventional pigment‐based coloring due to their excellent optical brightness, environmental sustainability, and remarkable long‐term stability [1, 2, 3]. In contrast to traditional dyes, which rely on molecular absorption and are affected by photobleaching, structural color originates from coherent scattering of light within ordered assemblies, providing intrinsic resistance to fading while imparting angle‐dependent optical behavior [2, 4, 5].

Colloidal self‐assembly has emerged as an effective strategy to fabricate photonic materials exhibiting structural colors, with ordered, densely packed arrays of polymeric or inorganic nanoparticles serving as basic building blocks [6]. When the periodicity of these arrays falls within the visible spectrum, the material exhibits a photonic bandgap and selectively reflects a specific wavelength of visible light, determining its observed structural color. Precise control over interparticle spacing, lattice symmetry, and refractive index contrast is crucial for tuning the wavelength of the reflected light [7, 8].

Hard particles, including inorganic ones, embedded in secondary matrices are usually used to generate colloid‐based structural colors [6, 9, 10, 11]. Due to their high refractive indices, they exhibit well‐defined interfaces and high reflectivity, resulting in vivid structural colors. However, in these systems, responsiveness and dynamic color tunability are typically introduced by modifications of the supporting matrix rather than through changes in the colloidal particles themselves [12, 13]. Moreover, the rigid colloids are often etched out in harsh postprocessing steps to obtain the structurally colored material [14, 15]. An elegant strategy to circumvent many limitations associated with the rigid particle‐based approaches is to develop soft colloidal systems, as they function both as reflecting particles and responsive building blocks. Of particular interest are low‐crosslinked microgels due to high control in synthesis, their broad application range, and intrinsic capability to self‐assemble into ordered assemblies under suitable conditions [16, 17, 18, 19]. The structural ordering is often transient, dynamic, and highly responsive to external stimuli [20]. Such responsiveness allows microgel‐based photonic structures to easily adjust their lattice spacing and, consequently, modulate in situ the reflected structural color. Additionally, due to their inherent compressibility, microgels offer an exceptional advantage: A single formulation of soft and deformable microgels can span a broad spectrum of structural colors simply through the adjustment of environmental parameters, including concentration, temperature, or pH [21]. Contrary to the use of hard particles, the inherent soft characteristics of microgels also induce greater tolerance for defects, allowing the formation of large‐scale colloidal assemblies [22].

Among stimuli‐responsive polymers, poly(N‐isopropylacrylamide) PNIPAm is the most widely used for the synthesis of thermoresponsive microgels [23, 24, 25]. PNIPAm‐based microgels can also be modified by the incorporation of secondary comonomers, introducing additional stimuli‐responsive behavior. At sufficiently high concentrations and after thermal annealing cycles across the volume phase transition temperature, PNIPAm‐based microgels, if appropriately designed, spontaneously form ordered structures exhibiting structural colors [26]. When fixed in a hydrogel matrix, such assemblies served specifically to monitor changes in temperature, pH, or metal ion concentrations in their environment [27, 28, 29, 30]. However, the formation of structural colors induced by fine‐tuning of the molecular factors in a large variety of stimuli‐responsive PNIPAm‐based microgels is still missing, which prevents an efficient selection thereof for desired applications.

**FIGURE 1:**
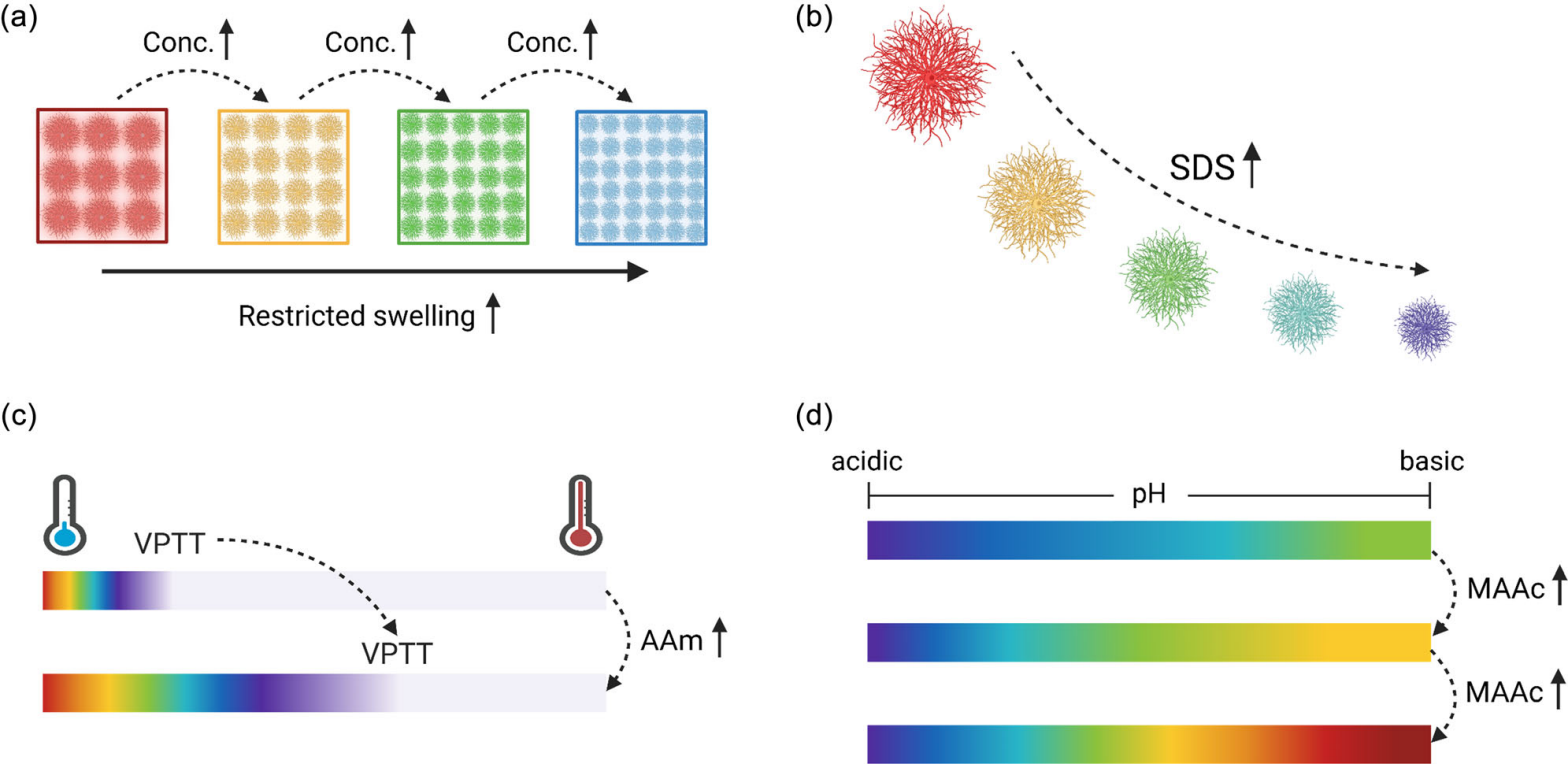
Schematic representations of the investigated microgel properties and their effect on structural color formation. (a) Microgel concentration modulation led to altered swelling behavior, resulting in a blueshift at higher concentrations. (b) Surfactant modulation enabled tuning of the microgel size and corresponding structural color. (c) Integration of a suitable monomer (acrylamide, AAm) allowed for the modulation of the “on/off” temperature switch for structural color. (d) Integration of a pH‐sensitive monomer (e.g., methacrylic acid, MAAc) modulated the response behavior of the structural color.

Here, we aim to bridge this gap by investigating large libraries of copolymer PNIPAm‐based microgels in which we vary different molecular components and conditions to obtain insight into their ability to induce structural color formation. We study how comonomer composition, particle size, and concentration influence the structural color formation and its tunability in thermally annealed PNIPAm‐based microgel colloidal systems (Figure 1a,b). Therefore, we chose three different comonomers to modulate the PNIPAm‐based microgels’ behavior in response to physical stimuli (temperature and pH). Using free‐radical precipitation polymerization, we synthesized (i) anionic microgels containing methacrylic acid (MAAc), (ii) cationic microgels containing 2‐(dimethylamino)ethyl methacrylate (DMAEMA), and (iii) neutral microgels containing acrylamide (AAm). MAAc and DMAEMA comonomers contain functional groups that impart (inverse) pH‐sensitive swelling behavior to the microgels [31, 32], while AAm was reported to modulate the volume phase transition temperature (VPTT) of PNIPAm [33].

In addition, by combining synthetic control, analysis of particle size and morphology, and thermal annealing into colloidal assemblies, we were interested in understanding the mechanisms of structural color generation and tunability in these soft copolymer materials [34, 35]. Detailed examination of their thermally induced and pH‐dependent swelling behavior, surface charge profiles, and dynamic assembly properties was necessary to get insight into the complex interplay between physicochemical properties and the optical output. Furthermore, we explored the possibility of tuning the observed reflection peaks upon environmental stimuli depending on the incorporated comonomer properties (Figure 1c,d).

Our study provides essential insights into the relationship between microgel composition, particle properties, concentration‐dependent ordering, response behavior, and resulting optical properties. This offers a foundation for designing dynamic, structurally colored colloidal photonic materials for a broad application range, including multifunctional sensors, optical tags, and responsive coatings.

## 2 Results and Discussion

### 2.1 Synthesis and Characterization of Microgel Libraries

**FIGURE 2:**
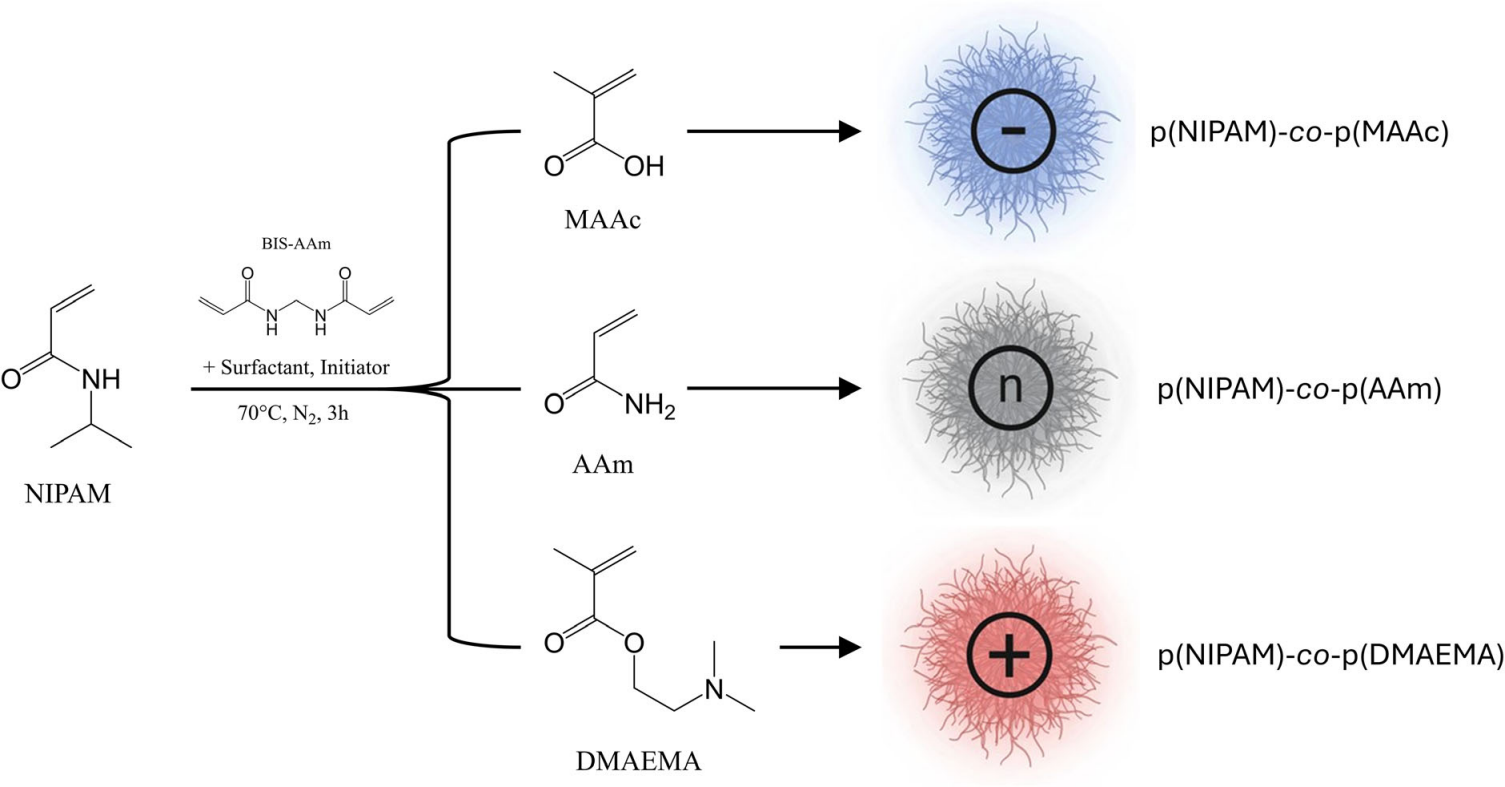
Chemical structures and synthetic approach used for the preparation of PNIPAm‐based microgels. Microgel size is precisely tuned by controlled addition of a surfactant during synthesis. Additional functionalities and responsiveness are conferred to PNIPAm‐based microgels by copolymerization with variable content of anionic (methacrylic acid, MAAc), neutral (acrylamide, AAm), or cationic (2‐(dimethylamino)ethyl methacrylate, DMAEMA) comonomers. The graphical representations of the microgels (right panel) are used for visualization purposes throughout the manuscript.

We utilized modified PNIPAm‐based microgels to serve as stimuli‐responsive colloidal building blocks for structurally colored materials. PNIPAm‐based microgels were synthesized via free‐radical precipitation polymerization and exhibited their characteristic thermally induced volume phase transition at a collapse temperature (VPTT) of approx. 32°C. Modification of the microgels was achieved by the incorporation of comonomers or employing surfactants during synthesis. Summary tables of these microgel libraries are provided in Tables S1–S6. To enhance the control over VPTT and introduce multistimuli responsiveness into PNIPAm microgels, we incorporated three different types of functional comonomers: acrylamide (AAm), methacrylic acid (MAAc), and 2‐(dimethylamino)ethyl methacrylate (DMAEMA) (Figure 2). AAm, a hydrophilic and nonionizable monomer, was chosen for its ability to form strong hydrogen bonds with water molecules, thereby increasing the strength of polymer–solvent interactions and consequently shifting the VPTT to higher temperatures [33]. MAAc and DMAEMA were incorporated to introduce pH‐dependent responsiveness to the resulting microgels through their respective (de)protonatable side chains [31, 32]. The comonomer additions yielded three types of microgels capable of sensing various environmental stimuli. For the intended application in photonic materials, we systematically optimized synthesis parameters, including comonomer content, initiator, and surfactant concentration, as well as solution pH, to obtain reproducible microgels with controlled size, narrow dispersity, and well‐defined responsiveness. This large library provides a basis for exploring how internal architecture and intrinsic morphology influence particle softness and crowding response and thereby the formation and tunability of structural colors, as well as their change in response to external stimuli.

A key structural feature for photonic assembly in concentrated dispersions is the internal particle architecture. PNIPAm microgels synthesized by precipitation polymerization are morphologically heterogeneous, with a densely cross‐linked core and a softer, polymer‐rich “fuzzy” corona [18, 23, 36, 37]. This radial stiffness gradient allows corona deformation and partial interpenetration under crowding, enabling effective volume fractions beyond hard‐sphere limits [38, 39]. In copolymer microgels, the partitioning of hydrophilic and/or ionizable comonomers during microgel growth can further lead to a functional‐group enrichment toward the periphery. For P(NIPAm‐*co*‐MAAc) microgels, a PNIPAm‐rich core with a PMAAc‐enriched outer region has been proposed to explain the broadened/weakened thermal transitions and the pronounced pH effects on collapse, via a charged, highly hydrated outer shell that constrains deswelling [40, 41]. Such core–shell mutual constraints can reshape the apparent transition profile and responsiveness [23, 42]. Under these conditions, crowding‐restricted reswelling reduces the center‐to‐center spacing after annealing, providing a physical basis for the concentration‐dependent shift of the Bragg reflection peak. Compression can also increase the polymer volume fraction and render the effective refractive index concentration‐dependent in strongly compressed regimes [43, 44, 45].

### 2.2 The Influence of Comonomer Type and Content on Microgel Responsiveness

First, we systematically evaluated the influence of comonomer content on the properties and responsiveness of each type of microgel. Changing the content of comonomer is expected to affect multiple microgel characteristics, including particle size, swelling response, and colloidal stability [23]. To ensure comparability, the surfactant concentration was kept constant within each microgel type and was specifically selected to create microgels with a suitable size range for use in structural color formation.

#### 2.2.1 pH‐Responsiveness of Anionic Microgels Containing MAAc Comonomer

A series of anionic microgels incorporating varying MAAc contents (0–25 mol%) was investigated to establish the effect of ionizable groups on microgel swelling behavior and responsiveness. As microgels containing a MAAc content above 25 mol% exhibited significant size polydispersity, they were excluded from further analysis, as monodispersity is essential for achieving structural colors with colloidal assemblies.

**FIGURE 3:**
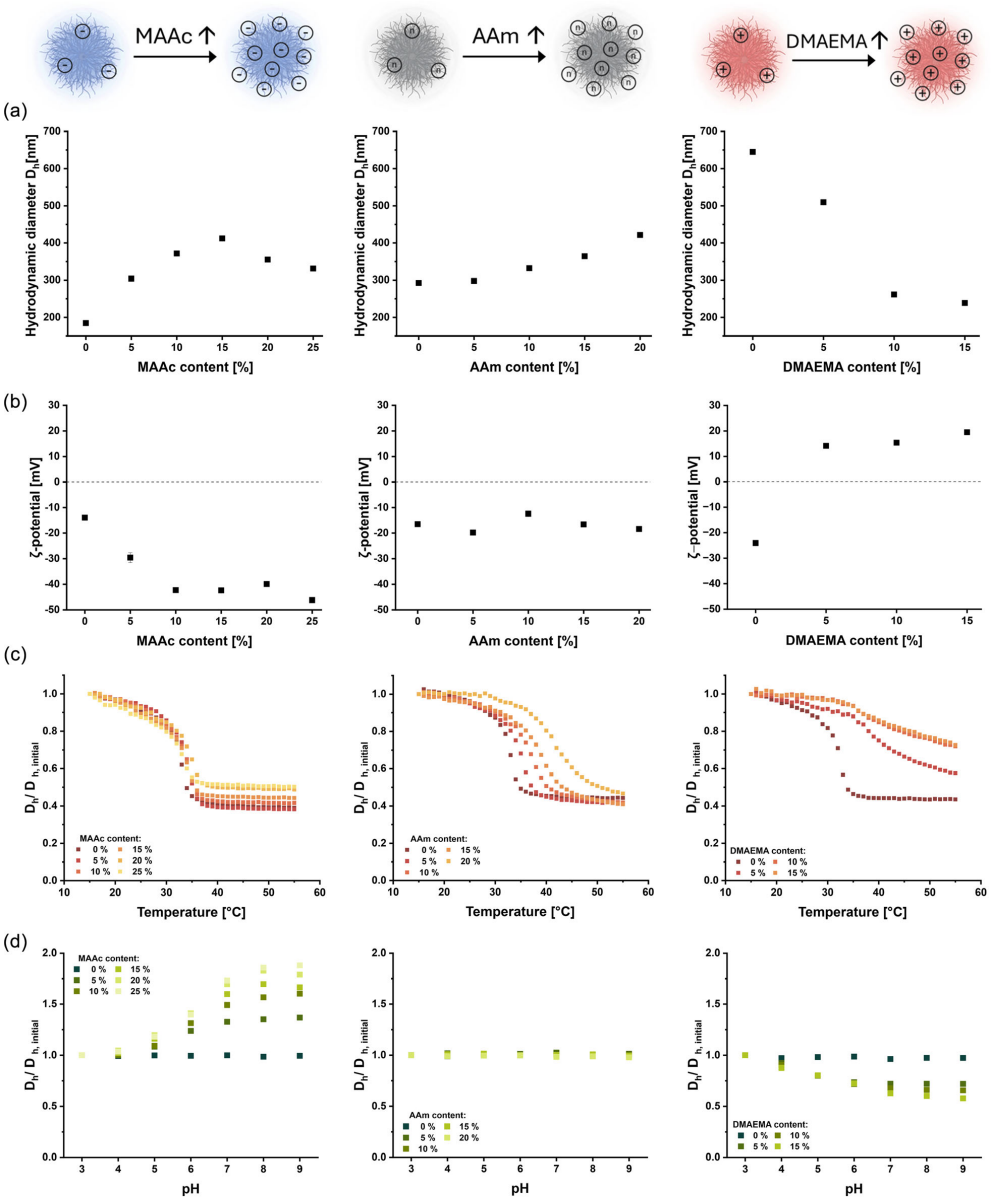
Dynamic light scattering characterization of PNIPAm‐based microgels synthesized using varying comonomer contents: MAAc (left column), AAm (middle column), and DMAEMA (right column). The surfactant concentration was kept constant within each microgel type. (a) Influence of the comonomer content on the average *D*_h_ of microgels. (b) Comonomer content‐dependent *ζ*‐potential measurements of microgels. (c) Temperature‐dependent swelling behavior of microgels illustrating the changes in size with the increase of temperature, normalized to the initial *D*_h_ at *T* = 15°C. (d) pH‐dependent swelling behavior of microgels indicating their responsiveness to acidic and basic environments, normalized to the initial *D*_h_ at pH = 3. Data points represent the mean value ± standard deviation (SD) of at least three independent measurements (*N* ≥ 3).

A clear relationship between microgel size and MAAc content was observed by dynamic light scattering (DLS). The smallest microgels, with an average hydrodynamic diameter (*D*_h_) of 185 ± 1 nm, were entirely composed of pure PNIPAm. With MAAc incorporation, particle diameter initially increased and reached a maximum average *D*_h_ of 412 ± 4 nm at 15 mol% MAAc before slightly decreasing at higher comonomer content (Figure 3a, left panel). The observed composition behavior likely reflects an equilibrium between the electrostatic repulsions among the charged polymer chains and the alterations in polymerization kinetics caused by the incorporation of comonomer [40]. At higher comonomer contents (>15 mol%), the excessive charge density may alter microgel nucleation, particle growth, and colloidal stability during synthesis, thus leading to smaller microgel sizes.

The *ζ*‐potential of microgels became progressively more negative with increasing MAAc content due to the incorporation of ionizable carboxylic acid groups, reaching −46.2 ± 0.9 mV at 25 mol% MAAc (Figure 3b, left panel). This enhanced surface charge density improves electrostatic stabilization, which is essential for colloidal stability and maintaining the long‐range order required for photonic applications.

Importantly, the responsive behavior of anionic microgels was significantly influenced by the MAAc content. With increasing MAAc content, a slight decrease in the VPTT was observed (31.3°C at 25 mol% MAAc), accompanied by a significant decrease in the overall shrinkage ratio at high comonomer concentrations, dropping to approximately 40% of the original relative size (Figure 3c, left panel). The diminished shrinkage reflects the decreasing fraction of thermoresponsive NIPAm repeating units within the microgel network capable of undergoing the volume phase transition.

In addition, the microgels exhibited a pronounced pH‐dependent swelling, yielding up to an 88% increase in size at basic pH compared to acidic conditions due to the electrostatic repulsions between the deprotonated MAAc units (Figure 3d, left panel). Since microgels composed entirely of PNIPAm lack pH‐responsiveness, this remarkable swelling behavior upon incorporation of MAAc highlights the benefits of using anionic microgels for dynamic modulation of interparticle spacing, thereby adding a second responsive feature for tuning the structural color of colloidal assemblies.

These findings are consistent with previous studies on PNIPAm microgels copolymerized with acrylic or methacrylic acid, where the introduction of carboxylic groups enhances the electrostatic stabilization and leads to a pronounced pH‐dependent swelling [40, 46]. In addition, our microgel library is providing an extensive set of systematic composition changes to explore their effect on microgel properties.

#### 2.2.2 VPPT Modulation of Neutral Microgels Containing AAm Comonomer

A second type of microgel, containing neutral AAm comonomers at varying concentrations up to 20 mol%, was synthesized. Microgels containing higher AAm contents (>20 mol%) exhibited significantly high size polydispersity and low synthetic yields, possibly due to the strong hydrophilic interactions of AAm interfering with microgel nucleation and particle growth.

The average *D*_h_ of microgels increased with the AAm content (from 293 ± 4 to 422 ± 2 nm), similar to the behavior observed for MAAc‐containing microgels (Figure 3a, middle panel). This size increase is attributed to the enhanced polymer solvation during nucleation and growth phases, as well as increased water uptake by the strongly hydrophilic AAm repeating units within the swollen microgel state [24].

The *ζ*‐potential of these neutral microgels remained slightly negative, attributed exclusively to the anionic moieties originating from the potassium persulfate (KPS) initiator (Figure 3b, middle panel). The slightly negative surface charge facilitated colloidal stability through electrostatic repulsion, thereby preventing microgel aggregation. Nevertheless, the *ζ*‐potential remained almost unaffected by varying AAm contents, consistent with the neutral, nonionizable character of the comonomer.

The incorporation of AAm significantly increased the VPTT, shifting it from 32.2°C for pure PNIPAm microgels to 41.8°C at 20 mol% AAm, without significantly affecting the relative magnitude of the thermally induced volume transition ratio (∼56% shrinkage, Figure 3c, middle panel). This upward shift in the VPTT may be explained by increased polymer–solvent interactions arising from the strong hydrophilicity of AAm repeating units, which effectively stabilizes the swollen microgel state and enhances the thermal energy required for deswelling [33]. Thus, the incorporation of AAm comonomer reveals a considerable potential as a strategy for fine‐tuning the VPTT of PNIPAm microgels, which is in agreement with previous reports on PNIPAm‐based microgels containing AAm comonomer [23, 47]. However, the pronounced AAm hydrophilicity imposes a practical limit on the achievable comonomer concentrations, as higher percentages of AAm compromise the microgel formation efficiency. The precipitation polymerization strategy used in this study relies upon reaction temperatures significantly above the VPTT of pure PNIPAm (≥70°C) to facilitate polymerization, microgel nucleation, and particle stabilization. Higher AAm contents may disrupt this balance due to the strong solvation effects. This finding highlights an upper limit for the successful integration of AAm into PNIPAm‐based microgels. Lastly, the neutral microgels displayed negligible pH‐responsiveness (Figure 3d, middle panel), confirming that the presence of AAm alone does not confer sensitivity to pH variations.

#### 2.2.3 pH‐Responsiveness of Cationic Microgels Containing DMAEMA Comonomer

The third type of synthesized microgels contained the cationic comonomer DMAEMA to achieve an inverse pH response compared to the anionic microgel type. Such cationic microgels are known to be particularly sensitive to synthesis conditions, including pH, ionic strength, and initiator type [48]. At higher fractions of cationic comonomer, they often exhibit lower yields and broader size distributions unless the polymerization medium is carefully controlled [49]. We successfully incorporated DMAEMA by altering the pH of the reaction mixture to 8 prior to initiation. Nevertheless, increasing the DMAEMA content over 10 mol% led to low synthesis yields (below 10%) and poor particle stability, indicating a substantial interference of the DMAEMA monomer in the polymerization process. Specifically, the incorporation of DMAEMA increases the overall hydrophilicity and introduces tertiary amine groups. These can interact electrostatically with the initiator KPS and form hydrogen bonds with neighboring NIPAm repeating units both intramolecularly and intermolecularly when protonated. Such interactions considerably restrict the polymer chain mobility, thereby interfering with the cooperative collapse of PNIPAm chains upon polymerization [48]. Moreover, the increased hydrophilicity imparted by the protonated amine functionalities stabilizes the swollen state of the microgels [49]. This effectively counteracts the thermally induced hydrophobic collapse characteristic of PNIPAm‐based systems necessary for colloid formation. Attempts at replacing the initiator with azo‐based alternatives like 2,2′‐azobis(2‐amidinopropane) dihydrochloride to reduce such interactions were unsuccessful due to the inactivity of the initiators at the required pH for DMAEMA integration [50].

While DMAEMA‐containing microgels were successfully synthesized, their lower yields indicate limited conversion and consequently smaller particle sizes at higher comonomer content (Figure 3a, right panel). The *D*_h_ of microgels dropped from 645 ± 7 nm for pure PNIPAm microgels to 238 ± 3 nm at 15 mol% DMAEMA. Comonomer incorporation into the microgels was confirmed by a clear shift in their *ζ*‐potential to positive values (average +16.3 ± 2.3 mV, Figure 3b, right panel).

The temperature response of the cationic microgels was drastically suppressed and the apparent VPTT shifted upward (up to 40.2°C; Figure 3c, right panel). In contrast to the sharp collapse typical of PNIPAm‐rich networks, the temperature‐induced deswelling transition of DMAEMA‐containing microgels became broadened and less pronounced. The size decrease at DMAEMA contents of 10 mol% or higher is gradual and reduced in magnitude compared with the anionic and neutral microgel series, reaching only a relative shrinkage of ∼20%. This decrease in size is likely due to the increased hydration and electrostatic/ion–dipole interactions associated with the tertiary amine groups of DMAEMA units, which counteract PNIPAm dehydration and hinder cooperative collapse, as well as a reduced polymerization efficiency and additional electrostatic interactions with the initiator KPS [49, 51, 52].

Despite these limitations, DMAEMA‐containing microgels exhibited clear and reproducible pH‐responsive behavior even at relatively low DMAEMA contents. A notable pH‐induced reduction in diameter of ∼27% was thus observed when shifting from acidic to basic pH conditions for microgels containing 5 mol% DMAEMA (Figure 3d, right panel). Increasing the DMAEMA content further enhanced this pH‐responsiveness, indicating tunability of the pH response behavior.

**FIGURE 4:**
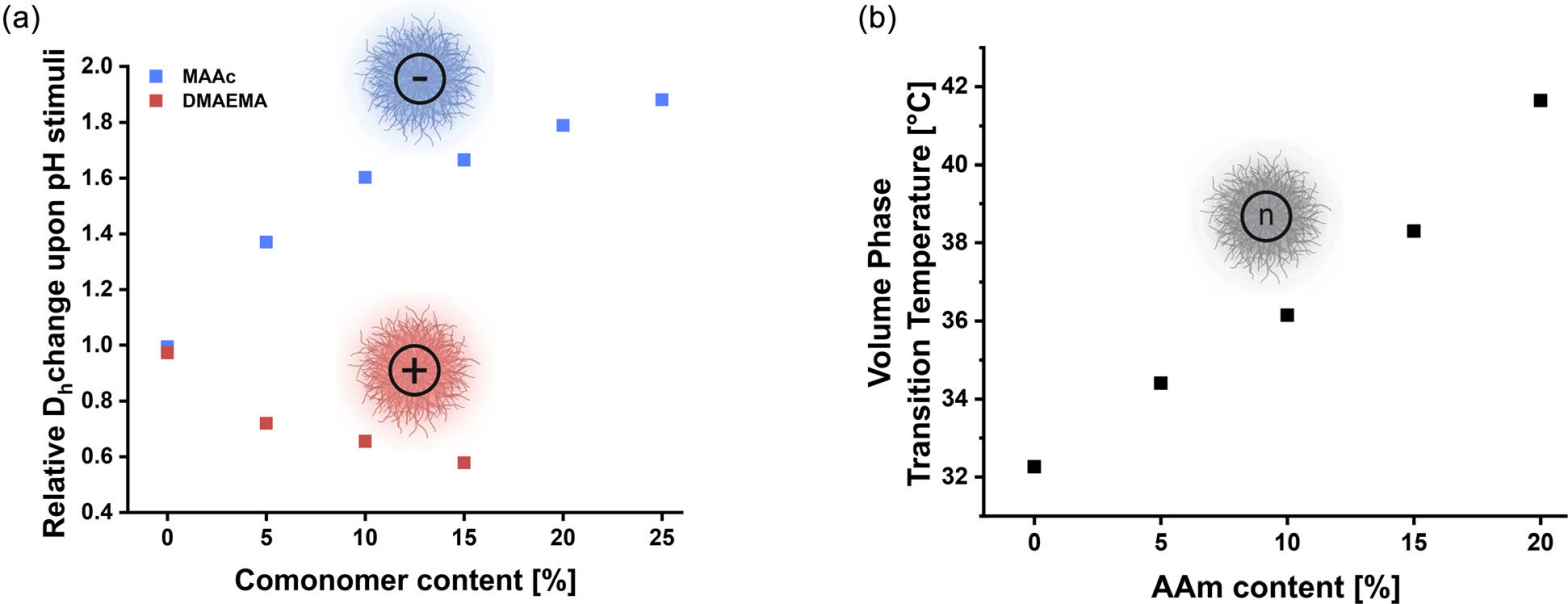
The effects of comonomer content variation for all microgel types. (a) Change in the pH response magnitude between acidic (pH 3) and basic (pH 9) conditions, depending on the comonomer type and content. (b) Modulation of the microgel VPTT depending on the AAm content.

**FIGURE 5:**
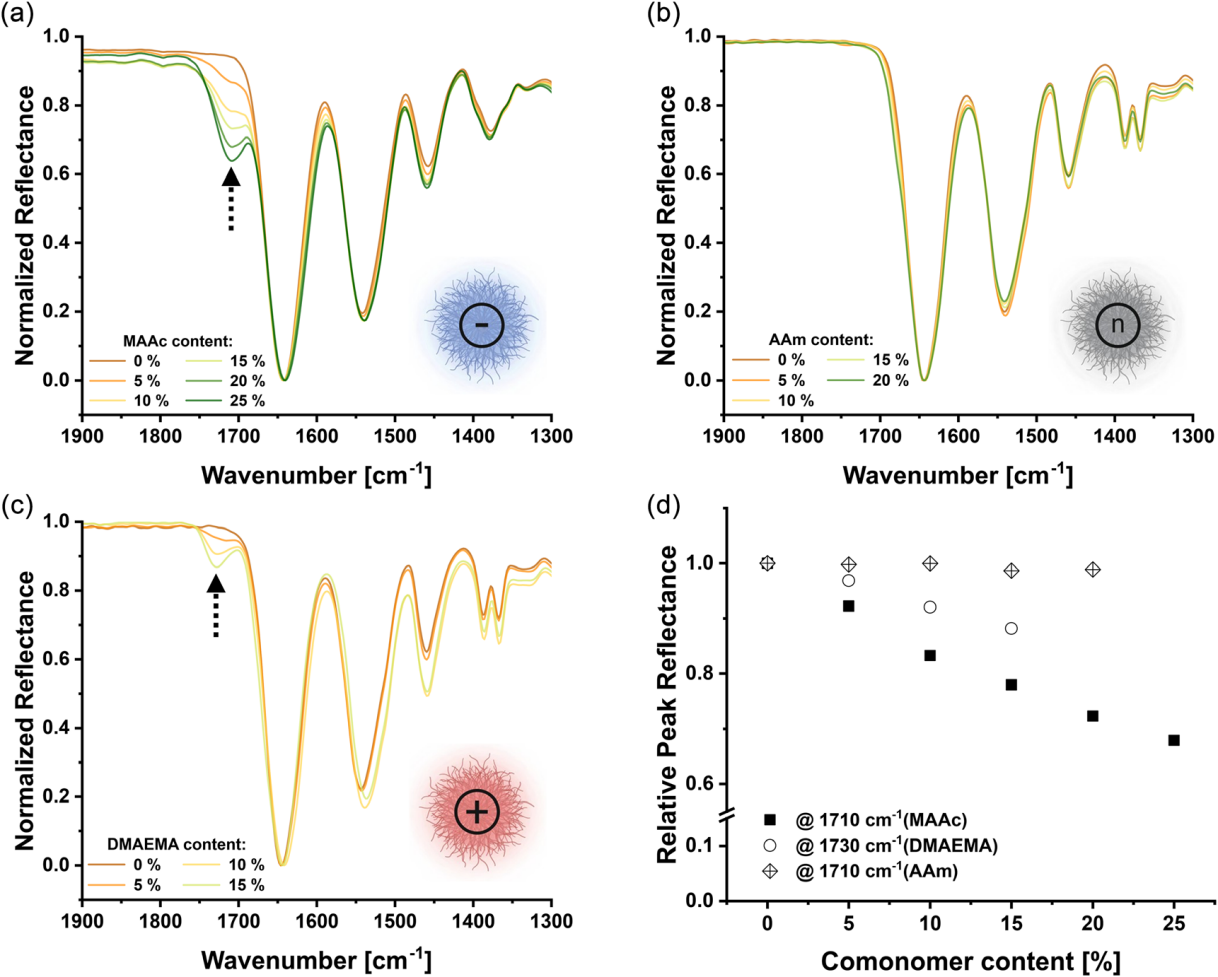
ATR‐FTIR spectra of freeze‐dried microgels with varying comonomer content. Fingerprint regions are shown for anionic (a), neutral (b), and cationic (c) microgels, normalized to the PNIPAm‐specific amide I band at 1640 cm^−1^ (see Figure S1 for full spectra). Dashed arrows indicate the comonomer‐specific bands used for semiquantitative evaluation. (d) Relative reflectance of the selected comonomer‐specific bands as a function of nominal comonomer feed.

As described above, key microgel properties were influenced by changes in comonomer content (Figure 4). The higher contents of pH‐responsive units (DMAEMA or MAAc) were correlated with larger relative changes in particle size upon pH variation. Similarly, the VPTT modulation upon incorporation of the neutral comonomer AAm became increasingly pronounced at higher comonomer contents. Furthermore, incorporation of the anionic and cationic comonomers was corroborated by the ATR‐FTIR spectra of freeze‐dried microgels, which show the appearance of bands specific for comonomers, which are absent in the spectrum of PNIPAm controls. After normalization to a PNIPAm reference band, the intensities of these bands increased linearly with the comonomer feed, allowing for a semiquantitative estimation of the incorporation efficiency and supporting the reproducibility of our libraries (Figure 5). For the neutral AAm series, no distinct bands were identified because its relevant vibrations overlap with those of PNIPAm. Nevertheless, the systematic shifts in VPTT and swelling behavior across the AAm‐containing microgels indicate the composition‐dependent change in polymer–solvent interactions. Taken together, these findings emphasize the complex interplay between chemical composition and internal polymer–polymer/polymer–solvent interactions and how they jointly modulate the resulting stimuli‐responsiveness.

### 2.3 The Influence of Surfactant on the Microgel Size

**FIGURE 6:**
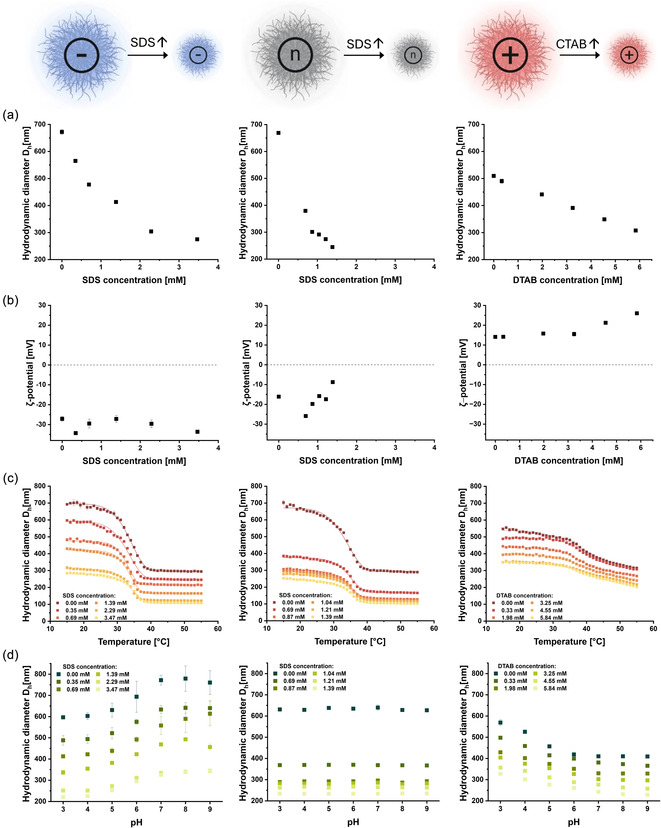
Dynamic light scattering analysis of PNIPAm‐based microgels containing a fixed comonomer content of 5 mol% (left: MAAc, middle: AAm, right: DMAEMA). Microgel size was precisely tuned via variation of surfactant concentration during synthesis, and their response behavior was systematically monitored. (a) Dependence of the hydrodynamic diameter (*D*_h_) on surfactant concentration. (b) *ζ*‐potential measurements as a function of surfactant concentration. (c) Temperature‐induced size response profiles of microgels with different diameters; solid lines represent sigmoidal fits to the data. (d) pH‐induced size responsiveness of microgels with various *D*_h_. All data are presented as mean value ± SD, obtained from at least three independent measurements (*N* ≥ 3).

The structural color exhibited by colloidal assemblies depends mainly on the size of the reflecting particles and their interparticle distance [7, 8]. Thus, achieving precise control over microgel size during synthesis was crucial to reliably tune their optical properties. Surfactants exert a direct influence on particle nucleation during polymerization by altering the interfacial tension, thus providing a straightforward and reliable method to tailor microgel sizes [36]. We systematically investigated the influence of surfactant concentration on particle size while keeping the comonomer content fixed at 5 mol% (Figure 6a). As preliminary experiments indicated that a hydrodynamic diameter (*D*_h_) range of approximately 250–350 nm was optimal for structural color applications, we targeted this size range and explored how surfactant concentration influenced both the resulting size of the microgels and their responsiveness. We selected the anionic surfactant SDS for microgels incorporating MAAc or AAm comonomers, while for DMAEMA‐containing cationic microgels, to whom SDS frequently caused aggregation, we selected the cationic surfactant dodecyltrimethylammonium bromide (DTAB). DTAB, with similar alkyl chain length and comparable critical micellar concentrations as SDS (8.3 mM for SDS and 15 mM for DTAB) [37], allowed direct comparison without detrimental aggregation effects.

The particle size of anionic microgels containing MAAc decreased with higher SDS concentrations, from 672 ± 9 nm (surfactant‐free) to 275 ± 4 nm at 3.47 mM SDS (Figure 6a, left panel). The polydispersity index (PDI) improved significantly from 0.07 in surfactant‐free conditions to 0.02 with surfactant addition, highlighting the enhanced monodispersity of microgels. However, at surfactant concentrations approaching the critical micellar concentration, particle growth was notably hindered, yielding microgels smaller than 100 nm with low yields. These very small microgel particles are of low interest for structural colors and were thus excluded from further analysis.

The particle size of neutral microgels containing AAm was similarly influenced by increasing concentrations of SDS, with a reduction of the *D*_h_ from 650 ± 6 nm (without surfactant) to 240 ± 3 nm at an SDS concentration of 1.39 mM (Figure 6a, middle panel). An excellent level of control and a low PDI of ≤0.1 were achieved. Interestingly, the surfactant‐induced size reduction effect was more pronounced in AAm‐containing microgels compared to MAAc‐containing microgels, likely due to the repulsive electrostatic interactions between the negatively charged groups of MAAc and SDS.

DTAB was successfully employed for size control and induced a size decrease of cationic microgels containing DMAEMA, from a *D*_h_ of 510 ± 3 nm in surfactant‐free conditions to 308 ± 3 nm at 5.84 mM DTAB (Figure 6a, right panel). While the cationic microgel size reduction was less pronounced compared to that of the anionic microgels, the target size range optimal for structural color formation was successfully achieved.

*ζ*‐potential values for all microgel types were consistent with their incorporated comonomer content. Specifically, anionic microgels exhibited strongly negative surface charges (average *ζ*‐potential of −30.2 ± 2.8 mV, Figure 6b, left panel), as expected from the deprotonated carboxyl groups. Neutral microgels were slightly negatively charged (average *ζ*‐potential of −17.3 ± 5.1 mV, Figure 6b, middle panel) due to the residual sulfate groups introduced by the initiator as explained above. In contrast, DMAEMA‐containing microgels showed distinct positive charges (average *ζ*‐potential of +17.8 ± 4.4 mV, Figure 6b, right panel). Importantly, the *ζ*‐potential values for all microgel types indicate that the surfactant concentration does not significantly alter the surface charge or interparticle electrostatic interactions. Controlling surface charge is crucial for applications in photonic materials, as altered surface properties could affect the packing behavior, lattice structure, and the resulting optical performance [53].

The thermal‐responsive property was not significantly affected by the surfactant concentration (Figure 6c). Considering that these microgels predominantly consist of NIPAm monomers (95 mol%), their characteristic volume phase transition remained stable and reproducible across all size ranges. For example, anionic microgels exhibited a consistent relative thermal shrinkage of approximately 58% ± 2% in *D*_h_, comparable to pure PNIPAm, with an average VPTT slightly elevated to around 33.3°C ± 0.4°C (Figure 6c, left panel). Similarly, neutral microgels exhibited a comparable relative shrinkage of 57% ± 1% at an average VPTT of 34.2°C ± 0.4°C (Figure 6c, middle panel). As described previously, the DMAEMA incorporation in cationic microgels partially suppressed their thermal response, reducing the relative temperature‐induced shrinkage to approximately 36% ± 2%. Additionally, their VPTT was elevated to 40.0°C ± 0.9°C, while making the transition less defined and more gradual (Figure 6c, right panel). Nevertheless, the reduction in response was reproducible across all microgel sizes.

Finally, the pH‐responsiveness remained consistent across all particle sizes for each microgel type, reflecting the fixed comonomer content of 5 mol% (Figure 6d). As expected, microgels containing AAm comonomers, lacking ionizable groups, showed negligible pH‐responsiveness (variation of about 0.1% ± 0.6% in *D*_h_) when transitioning from acidic to basic conditions (Figure 6d, middle panel). Anionic microgels displayed swelling (∼39% ± 10% increase in *D*_h_) at basic pH, driven by electrostatic repulsion among negatively charged carboxylate groups (Figure 6d, left panel). Inversely, the DMAEMA side chains in the cationic microgels induced swelling at low pH due to their positively charged tertiary amine moieties. At higher pH, the uncharged state promoted a more compact structure: Cationic microgels shrunk by approximately 25% ± 2% (Figure 6d, right panel), resulting from deprotonation and loss of electrostatic repulsion among the tertiary amine groups. The reduced magnitude of pH response further supports the hypothesis of lower DMAEMA incorporation efficiency mentioned previously.

The systematic variation of parameters for microgel synthesis allowed effective control and fine‐tuning of both particle size and responsiveness to stimuli as a prerequisite for the use of these microgels in structurally colored materials.

### 2.4 Formation of Structurally Colored Microgel Materials and Their Application

**FIGURE 7:**
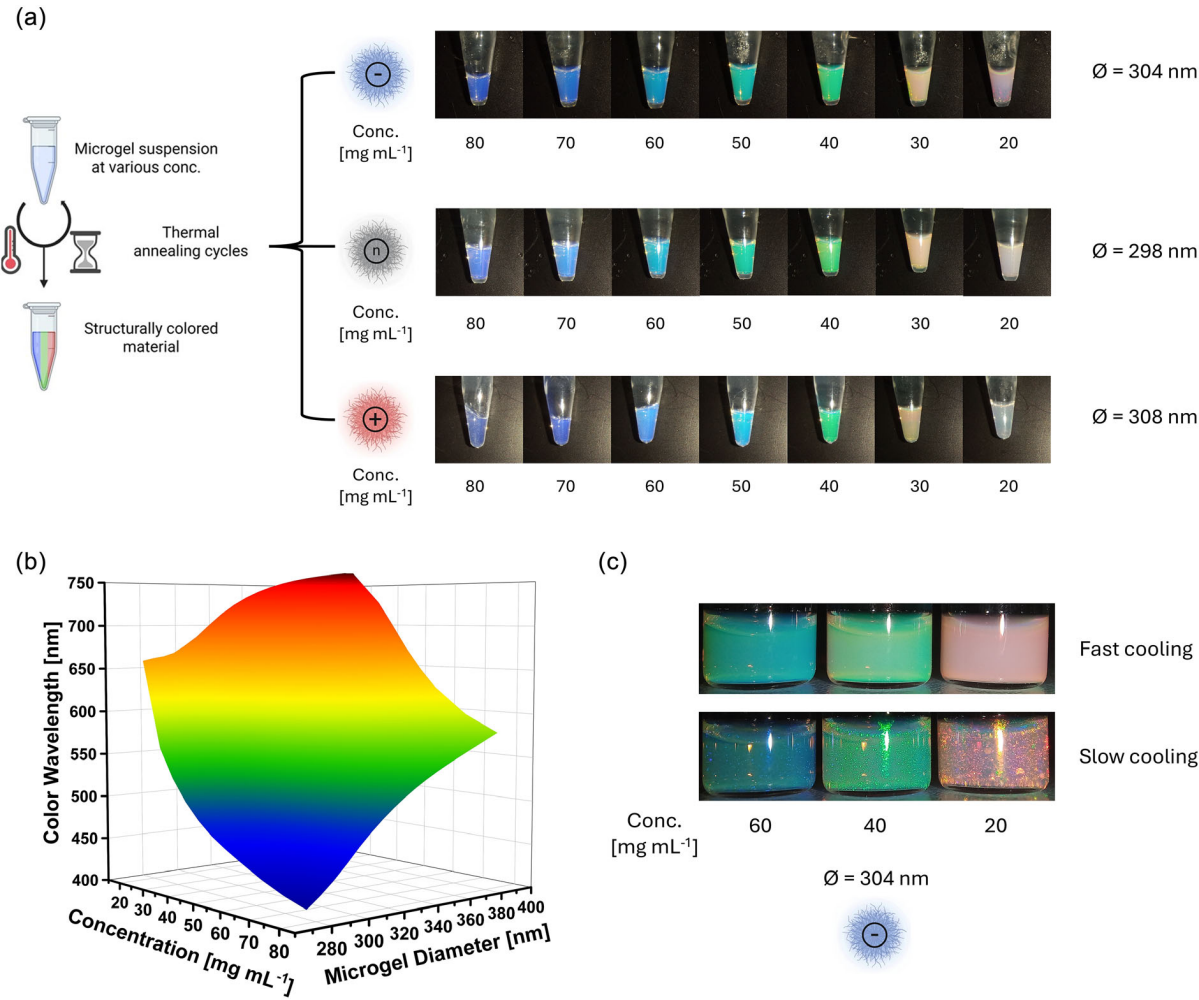
(a) Formation of structurally colored materials by thermally assisted self‐organization of microgels containing 5 mol% comonomer. Repeated thermal cycling across the VPTT (up to five times) promotes ordering at sufficiently high concentrations (>20 mg mL^−1^), allowing for regular photonic assemblies. The representative images (right panel) illustrate that the packing spacing, and thus the reflected color, can be tuned by concentration, covering the visible range. (b) Design map highlighting the experimentally accessed parameter space (hydrodynamic diameter and concentration of microgels) and the corresponding reflectance peak positions (*λ*_max_) measured in this study. The plotted colors approximate the observed structural colors. (c) Exemplary images of anionic assemblies comparing fast versus slow cooling during thermal annealing, with slow cooling leading to more ordered, angle‐dependent, iridescent assemblies, consistent with the improved formation of crystalline domains.

We investigated the self‐organization method to form structural colors by regularly arranging the different types of microgels. Self‐organization occurred by rehydration of the microgels in Milli‐Q water at sufficiently high concentrations and was facilitated by additional thermal cycling across the VPTT (Figure 7a, left panel) [21]. Importantly, the anionic and neutral microgels showed no heating/cooling hysteresis, indicating reproducible, near‐equilibrium collapse–reswelling cycles during the thermal annealing process (Figure S2). For the cationic (DMAEMA‐containing) microgels, reswelling upon cooling was slightly delayed, consistent with their broadened and attenuated thermal transition. However, the fully collapsed and fully swollen endpoints remained unchanged, confirming the reversible thermoresponsive behavior over the investigated temperature range. By repeatedly collapsing and reswelling the particles up to five times, the microgels eventually organized in a regular arrangement. The tight packing was observable by scanning electron microscopy (SEM), although distinct particles were only observable in the collapsed state of the microgels (Figure S3, right micrograph compared to left and middle micrographs). While qualitative, these results indicate that the structural color reflects the tight packing of particles in the assembled state.

We achieved structurally colored microgel suspensions for all types of microgels. The formed structural color was strongly influenced by the interparticle distance the microgels equilibrate to after the thermal cycles. A highly crowded environment does not allow full reswelling and leads to tighter packing, while the microgels equilibrate further apart at low concentrations. An advantage of this approach is that the structural color can be predictably selected across the visible spectrum by choosing the appropriate combination of microgel size and concentration from this library of microgels (Figure 7a, right panel). The design map presents the experimentally accessible parameter range (i.e., microgel hydrodynamic diameter and concentration) and the corresponding reflected peak wavelengths (Figure 7b). This plot provides a direct overview of the *D*_h_ range and concentration range over which structural color is obtained in our microgel systems and visualizes how the reflected wavelength shifts across the visible spectrum as these variables are tuned.

**FIGURE 8:**
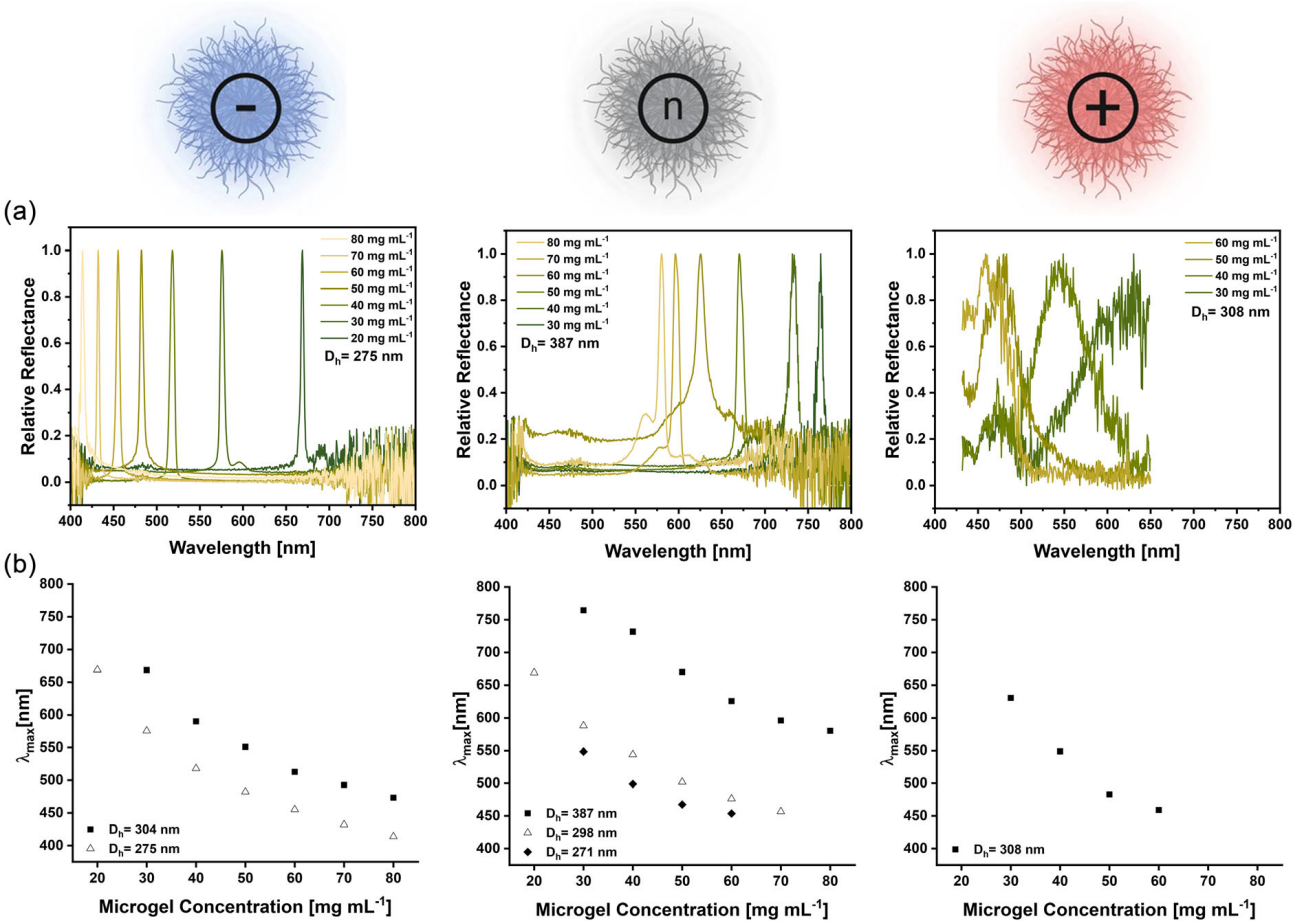
Reflectometry measurements of structural colors formed through self‐organization of microgels containing 5 mol% of comonomers. (a) Exemplary normalized spectra of structurally colored suspensions at different microgel concentrations. The hydrodynamic diameter (*D*_h_) of the microgels is indicated within the graph. If needed, excessive noise regions were removed for clarity. (b) Peak wavelength (*λ*_max_) extracted from the reflectance spectra as a function of microgel concentration.

When *D*_h_ remained constant, the measured peak reflection wavelength was approximately inversely dependent on the concentration of microgels in suspension for each of the microgel sizes (Figure 8a). However, the peak wavelengths at similar concentrations of microgels were shifted by at least twice the difference in diameter (Figure 8b). As an example, the tested anionic microgels have a difference in diameter of Δ*D*_h_ = 29 nm, while their peak wavelength difference Δ*λ*_max_ is always greater than 60 nm. The concentration‐dependent reflectance trends indicate that despite their softness and deformability, PNIPAm‐based microgels can reproducibly form photonic assemblies over a broad design range of particle concentration (20–80 mg mL^−1^) and size (approx. 270–390 nm), especially for the anionic and neutral series. For microgels with comparable hydrodynamic diameters and similar thermoresponsive collapse behavior, the concentration‐dependent color shifts are relatively consistent. This indicates that, in the high concentration regime, the optical response is governed mainly by crowding‐controlled packing rather than composition or long‐range interactions. As microgels cannot fully reswell upon cooling at high concentrations, they adapt to each other through compression, corona deformation, and partial interpenetration. These short‐range packing constraints determine the center‐to‐center spacing and thus the reflected wavelength. In contrast, comonomer chemistry and *ζ*‐potential are expected to influence the degree of order (defect density, peak intensity, and bandwidth) and the annealing kinetics. However, the DMAEMA‐containing microgels deviate from this trend, consistent with their altered collapse behavior (see below).

When the cooling step of the thermal annealing cycle to room temperature was extended to 6 h, the anionic and neutral microgels formed colloidal crystals with iridescent structural colors. Images of larger‐volume microgel dispersions visualize macroscopic crystal‐like domains and their angle‐dependent iridescence and concentration‐dependent color evolution (Figure 7c). The reflectance spectra of these samples showed clear and intense Bragg peaks (Figure 8a, left and middle panels). A summary of the corresponding spectra, including the non‐normalized peak intensities and full width at half maximum (FWHM) values, is provided in Table S7.

By contrast, the cationic microgels produced concentration‐dependent hues but did not form colloidal crystals under the thermal annealing conditions used here. This behavior is mainly due to their reduced and broadened thermoresponsive transition, which is consistent with the attractive interactions introduced by DMAEMA. Its protonated tertiary amine groups can interact intra‐ and intermolecularly with the negatively charged sulfate groups derived from the KPS initiator. These interactions act as ionic cross‐links, restricting chain mobility and counteracting the cooperative collapse of the PNIPAm‐rich network. Transmission electron microscopy (TEM) provides further qualitative support for this interpretation by showing a distinct morphology for the cationic microgels (Figure S4). They appear less uniformly spherical, with a higher size distribution and a strong tendency for aggregation compared to the anionic and neutral analogs at comparable comonomer content.

The effective “annealing window” for rearrangement and defect correction during repeated collapse/reswelling cycles is expected to narrow under these conditions. Consequently, this suppresses long‐range ordering and favors kinetically trapped, gel‐like, or amorphous microgel packings at the high concentrations required for Bragg reflection instead of long‐range crystalline order. Such structural heterogeneity also increases scattering and reduces optical coherence, consistent with the low peak intensities (<10%) and high noise observed for the cationic assemblies (Figure 8a, right panel). Therefore, the cationic series was not further used to perform stimulus‐dependent peak‐shift experiments, as these measurements require colloidal crystals with well‐defined stop bands to quantify reversible color changes reliably.

To gain further insights into the structure factor of microgel assemblies, static light scattering (SLS) was performed. SLS measurements of microgel suspensions at low microgel concentrations (<10 mg mL^−1^) reveal regular particle arrangements, with evidence of a body‐centered cubic (BCC) crystal structure formation [54] for anionic and neutral microgels and tight packing without long‐range order for cationic microgels (Figure S5). However, in the concentration regime required for inducing structural color (20–60 mg mL^−1^), excessive multiple scattering events render the method unsuitable. Further investigations into the structural arrangements of the swollen microgels at high concentration would require contrast‐engineered particles with dense cores [55], which does not preserve the intrinsic soft nature of our system, or synchrotron‐grade diffraction experiments (X‐ray or neutron), which are beyond the scope of this study [56].

#### 2.4.1 Structural Color Change Through Environmental Stimuli

First, we explored the changes in structural colors upon changes in temperature for neutral microgels, as they presented the most significant change in thermo‐responsive behavior depending on comonomer content. Generally, by increasing the temperature above the VPTT of the microgels, the structural color is lost due to the collapse of the particle, as well as the disassembly of the colloidal arrangements [57]. The result is a highly scattering, milky‐white suspension. As described above, if such suspensions are cooled below the VPTT, the structural color is restored accordingly; hence, the temperature can be considered an “on/off” switch [58].

**FIGURE 9:**
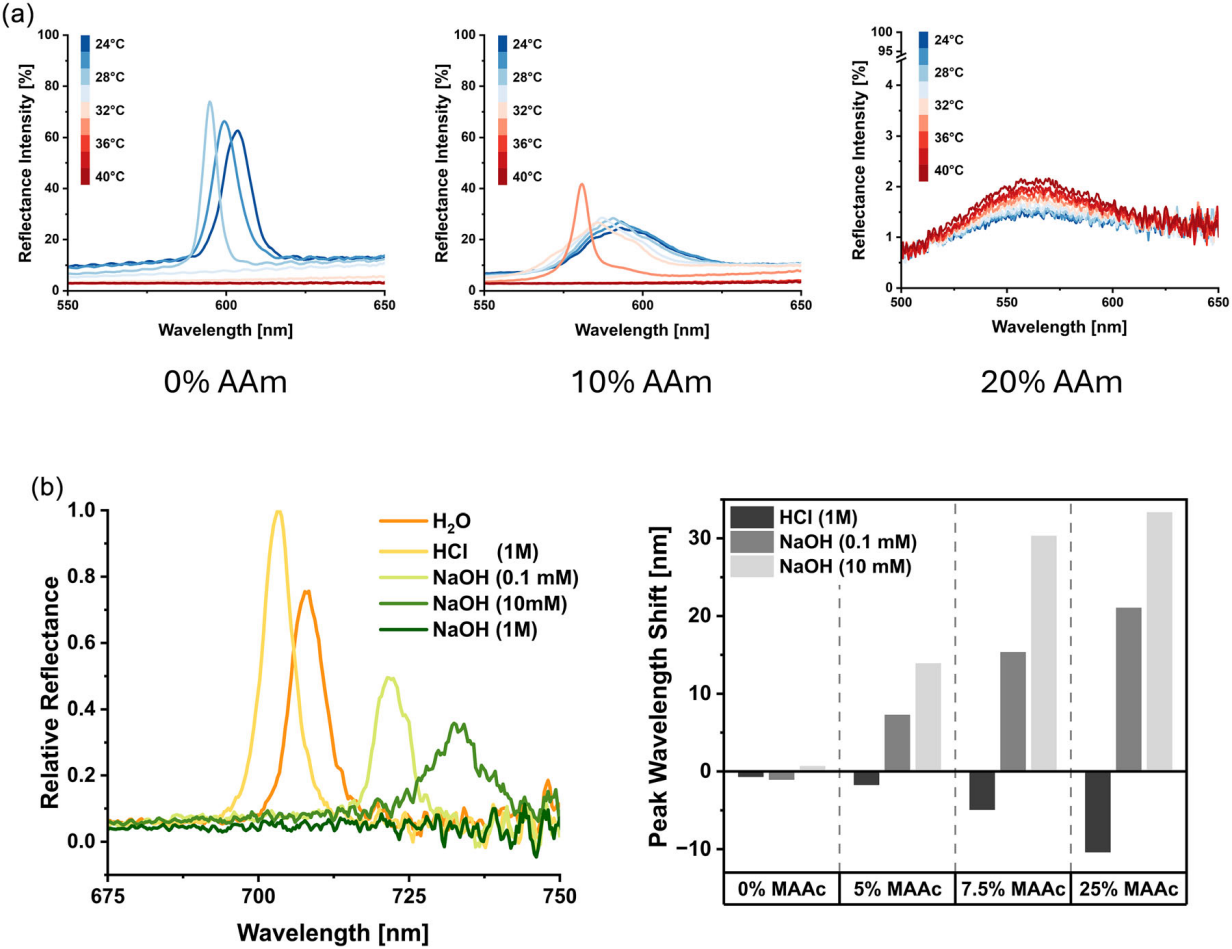
Characterization of microgel‐based structural colors under different environmental stimuli by reflectometry. (a) Colored microgel suspensions containing different AAm comonomer percentages are exposed to increasing temperatures. Corresponding reflection spectra are depicted in a temperature‐dependent manner (24°C–40°C, steps of 2°C). (b) Colored microgel suspensions containing different MAAc comonomer percentages were exposed to various acid and base concentrations. Left: exemplary reflectometry spectra of microgel suspensions containing 7.5 mol% MAAc. Right: peak reflectance wavelength shifts of microgels containing various percentages of MAAc, compared to their respective water control.

We established previously that we can tune the VPTT of neutral microgels by increasing the AAm comonomer content, which shifted it to higher values (Figure 4b). Hence, we explored the tuning of the “on/off” point of their structural colors (Figure 9a). For microgels containing no AAm comonomer, the structural color strongly changed with the increase of temperature up to 30°C: A blueshift and a narrowing of the reflection peak were observed, corresponding to the partial collapse of particles and resulting increase of the refractive index of the microgels in suspension. As expected, above 30°C, the structural color and reflection peak disappeared. This temperature range agrees with the start of the volume phase transition characterized using DLS. A similar behavior was observed for microgels containing 10% AAm comonomer; however, the transition was shifted to higher temperatures by approximately 6°C. Lastly, while microgels containing 20% AAm comonomers did exhibit structural colors, the reflection peak was of low intensity (Figure 9a, right panel). We observed that at this AAm comonomer content, the resulting microgels did not form colloidal crystals, likely due to increased interparticle interactions through hydrogen bonding of the AAm comonomer, preventing crystallization. Nevertheless, the observed structural color was still visible at 40°C, supporting the tuning of the “on/off” switch of microgel‐based structural colors using increased percentages of the AAm comonomers.

Second, we used the anionic microgels to investigate the structural color change to variation of pH. Microgels containing different percentages of MAAc comonomers were thermally annealed to form structurally colored suspensions. Afterward, different concentrations of HCl and NaOH were added to induce a change of the solution pH and microgel size, thus shifting the structural color reflection peak (Figure 9b). To account for dilution effects, the spectra were referenced to a control sample supplemented with the same volume of water. While the addition of strong acids/bases disrupted the colloidal crystals, they were reformed after a thermal annealing cycle, and the reflection peaks were shifted according to the change in microgel dimensions. Acidic conditions blueshifted the reflection peaks, while basic conditions led to a redshift, in agreement with the measured diameter changes observed in DLS measurements (Figure 4a). Similarly, the magnitude of the response was increased at higher MAAc comonomer content, while pure PNIPAm microgels were not affected (Figure 9b, right panel). The strongest basic conditions tested (1 M NaOH) led to the complete loss of structural color, as the anionic microgels likely started to degrade. Contrarily, for the different concentrations of base (0.1 and 10 mM NaOH), swelling of the anionic microgels and thus color shifts were observed. Additionally, a clear correlation between the base concentration and the peak wavelength shift was observed, indicating the possibility of sensing pH changes using our microgel‐based structural colors. While absolute determination of pH was not possible due to the low sample volume and high viscosity, the expected pH trends were still observed when equally diluting the samples to measurable conditions (Table S8).

Interestingly, the peak wavelength shift in strong acidic conditions was less pronounced than for weak basic conditions. Presumably, this is due to the microgels already being mostly collapsed at high concentrations of particles, as well as the generally acidic environment in the high concentration microgel suspensions originating from the sulfonate end groups of the initiator KPS. Nevertheless, the observed blueshift is clearly dependent on the comonomer content of the microgels. While forming structural colors, cationic microgels were not investigated in terms of their change in reflectance, as their different morphology prevented clear reflection peaks. The addition of acids/bases also changes the ionic strength of the solution, which is known to affect microgel swelling under dilute conditions [59]. Consistent with this, our MAAc‐containing microgels showed a measurable decrease in diameter by DLS upon addition of NaCl. However, the corresponding reflectance peak shifts in concentrated colloidal assemblies upon NaCl addition were too small, without a clear dependence on microgel composition, and well below the pH‐induced shifts (Figure S6). This is likely due to the high particle concentration regime where structural color develops, when crowding and confinement dominate the packing of microgels and strongly limit further deswelling induced by ionic strength, thereby attenuating the optical response of microgel dispersions.

## 3 Conclusion

In this study we demonstrate high control over the synthesis of PNIPAm‐based microgels as stimuli‐responsive materials with dynamic structural colors. The incorporation of neutral (AAm), anionic (MAAc), and cationic (DMAEMA) comonomers into copolymer microgels led to the desired response behavior. AAm‐containing microgels showed an increase in VPTT, while MAAc‐ and DMAEMA‐containing microgels showed excellent (inverse) response behavior to pH changes in addition to their intrinsic thermal response. For all types, we finely tuned the magnitude of the property change by varying the respective comonomer content.

Additionally, we utilized surfactants to precisely control the size of such copolymer microgels. This is crucial for the proposed application in structural colors, where low polydispersity and tailored particle sizes are required. Optimal microgel sizes for the visible wavelength range (250–350 nm in diameter) were successfully achieved. By thermally annealing concentrated microgel suspensions, colloidal assemblies with structural colors were generated, independent of the microgel type. Furthermore, for anionic and neutral microgels, iridescent colloidal crystals were obtained through slow cooling to room temperature. Cationic microgels did not form colloidal crystals, as they exhibited increased interparticle interactions and a limited thermal response.

We found the produced structural colors to be directly dependent on both microgel diameter as well as concentration. In highly concentrated suspensions, full microgel swelling was prevented, which led to shorter interparticle distances and a blueshifted reflection peak. Inversely, low concentrations allowed for full swelling and a redshifted color. Furthermore, we manipulated the reflected color wavelength through environmental stimuli. We found that the change in VPTT in AAm‐containing microgels led to an increase in color stability at higher temperatures. Hence, we were able to tune the “on/off” switch for structural color. Similarly, we were able to control the magnitude of the peak wavelength shift upon pH change by employing microgels containing different MAAc comonomer contents, which correspond well to the expected swelling properties of the microgels.

In summary, we demonstrated a high degree of control over material preparation and response properties required when using soft colloidal assemblies. We believe our findings lay the groundwork for a systematic approach to using NIPAm‐based copolymer microgels as colloidal building blocks in structural color applications such as sensor devices, coating materials, and optical tags. Importantly, these microgels represent a highly versatile and reproducible platform for constructing soft colloidal materials capable of displaying dynamically tunable structural colors.

## 4 Experimental Methods

### 4.1 Materials

*N*‐Isopropylacrylamide (NIPAm, 97%), methacrylic acid (MAAc, 99%), *N*,*N*′‐methylene bis(acrylamide) (BIS, 99%), sodium dodecyl sulfate (SDS, 99%), potassium persulfate (KPS, 99%), acrylamide (AAm, 99%), 2‐(dimethylamino)ethyl methacrylate (DMAEMA, 98%), dodecyltrimethylammonium bromide (DTAB, 98%), NaOH, and HCl were purchased from Sigma–Aldrich and used as received, if not mentioned otherwise. NIPAm was recrystallized from hexane prior to use. All experiments were performed using deionized water from a Milli‐Q water purification system (Millipore, resistivity of 18.2 MΩ cm^−1^) fitted with a 450 µm filter.

### 4.2 Synthesis of Microgels

Microgels were synthesized via free‐radical precipitation polymerization, following a protocol adapted from literature [60]. Briefly, the reaction was carried out in a 100 mL three‐neck round‐bottom flask equipped with a reflux condenser, thermometer, inlet for inert gas (nitrogen), and a magnetic stirrer. Appropriate amounts of NIPAm, selected comonomer (MAAc, AAm, or DMAEMA), BIS (2.5 mol% with respect to total monomer content), and surfactant (SDS for anionic/neutral and DTAB for cationic microgel systems) were dissolved in water (48 mL). The total monomer content was fixed at 5.3 mmol for all reactions. For DMAEMA‐containing microgels, the pH was adjusted to 8 using HCl (0.5 M) to improve the incorporation of comonomer.

The reaction mixture was stirred at 300 rpm and degassed under N_2_ for 1 h, while heating to 70°C. Polymerization was initiated by rapid addition (in one shot) of aqueous KPS solution (2 mL, 1 mol% relative to total monomer content), previously degassed under N_2_ for 15 min. The reaction proceeded for 3 h under inert atmosphere and was quenched by exposure to air, followed by cooling to room temperature. The resulting microgels were purified by four cycles of centrifugation and redispersion in deionized water. Centrifugation speed and duration parameters were optimized for the microgel properties to ensure efficient purification. Microgels were stored as aqueous stock suspensions. When dry microgels were required, aliquots were frozen in liquid N_2_, freeze‐dried for 72 h in an Alpha 2‐4 LD Plus lyophilizer (Christ), and stored under vacuum.

### 4.3 Characterization of Microgels

DLS and zeta‐potential (*ζ*‐potential) measurements were conducted using a Zetasizer Nano ZsP (Malvern Instruments) on dilute microgel suspensions (∼0.1 mg mL^−1^). All samples were equilibrated at 20°C for 180 s prior to measurements, and the data were collected using a back‐scattering angle of 173°. The hydrodynamic diameters (*Z*‐averaged *D*_h_) and *ζ*‐potential values were reported as the average from at least three independent measurements. The error bars depict the standard deviation. The PDI values were determined using the instrument's accompanying analysis software.

The thermoresponsive behavior was characterized by recording DLS data at temperatures ranging from 15°C to 55°C in steps of 1°C, allowing 10‐min equilibration time at each step. The VPTT was extracted by fitting the temperature dependence of the DLS data with a Boltzmann sigmoidal model and defining the VPTT as the midpoint value of the fit, which provides an excellent fit quality for all microgel samples (*R* ^2^ ≥ 0.99) [61]. The pH‐responsiveness was assessed by titrating the dilute microgel suspensions with NaOH or HCl (0.25 M each) to adjust the pH between 3 and 9. Microgel samples were allowed to equilibrate at room temperature for 24 h before the DLS measurements were performed as described above.

### 4.4 Attenuated Total Reflectance Fourier Transform Infrared Spectroscopy

Attenuated total reflectance Fourier transform infrared (ATR‐FTIR) spectroscopy measurements of freeze‐dried samples of microgels containing various comonomer contents of each type were performed on an Alpha II Platinum ATR‐FTIR (Bruker). Spectra were measured between 4000 and 400 cm^−1^, at a resolution of 4 cm^−1^, with 24 scans per sample. The resulting spectra were smoothed, baseline/H_2_O/CO_2_‐corrected and normalized to the amide I band (C=O) at 1640 cm^−1^ characteristic for NIPAM. The incorporation of comonomer was estimated by comparison of the carboxylic acid C=O band at 1710 cm^−1^ for MAAc and the ester C=O band at 1730 cm^−1^ for DMAEMA. For AAm, no characteristic bands were identified.

### 4.5 Preparation of Structurally Colored Colloidal Microgels

Structurally colored microgel materials were fabricated via the colloidal self‐assembly approach. For this, lyophilized microgels were rehydrated in deionized water at a concentration of 80 mg mL^−1^, subjected to thermal annealing (50°C for 1 h), followed by cooling to room temperature overnight. To ensure complete solvation and assembly, thermal annealing was repeated up to 5× until colloidal crystal formation. Evaporation of water was prevented by using O‐ring sealed tubes and parafilm. These stock solutions were diluted appropriately for lower microgel concentrations and thermally cycled to reform colloidal crystals.

### 4.6 Reflectometry

Optical reflectance spectra were obtained using a bifurcated fiber optic backscattering probe (R200‐7‐SR, Ocean Optics) coupled to a 6200K white LED light source (EvoluChem) and an AvaSpec‐3648 spectrometer (Avantes). Reflectometry measurements and response behavior experiments were performed on 50 µL aliquots in PCR tubes. The spectra were recorded by resting the probe flatly onto samples and normalized to the peak reflection intensity when appropriate.

### 4.7 Color Shift Upon Environmental Stimulation

To observe the modulation of VPTT and its effect on structural colors, colloidal crystal suspensions of AAm‐containing microgels at a concentration of 40 mg mL^−1^ were heated on a hotplate in increments of 2°C. Time‐resolved experiments showed a temperature‐induced response within a few seconds upon heating (Figure S7). Nevertheless, the reflection spectrum was recorded after an equilibration time of 5 min.

For the color shift as a response to pH changes, suspensions of MAAc‐containing microgels (40 mg mL^−1^) were supplemented with 5 µL of either HCl (1 M) or NaOH (1 M, 10 mM, or 0.1 mM). As a control, one sample was supplemented with 5 µL of water to account for concentration changes. Reflection spectra and peaks were then compared to this control sample to identify peak wavelength shifts and determine the pH‐dependent response behavior isolated from potential dilution effects.

### 4.8 Statistical Analysis

DLS data (*D*_h_ and *ζ*‐potential) were reported as mean values from at least three independent measurements (*N* ≥ 3), and the error bars depict the corresponding standard deviation (SD).

## Supporting Information

Additional supporting information can be found online in the Supporting Information section. **Supporting Fig. S1:** ATR‐FTIR spectra of microgels containing varying comonomer contents, normalized to the amide I (C=O) stretching band at 1640 cm^−1^. **Supporting Fig. S2:** Normalized DLS temperature‐induced size response profiles of PNIPAm‐based microgels containing 5 mol% comonomer (left: MAAc; middle: AAm; right: DMAEMA), recorded during consecutive heating (red dots) and cooling (blue dots) from 15°C to 55°C. **Supporting Fig. S3:** Electron microscopy micrographs of anionic microgel assemblies (5 mol% MAAc, *D*_h_ = 304 nm) in the indicated state. (Left) Cryo‐SEM micrograph of a flash‐frozen microgel assembly. (Middle) SEM micrograph of a lyophilized microgel assembly. (Right) SEM micrograph of a microgel assembly fixed and dried in a cross‐linked secondary matrix to preserve the particle arrangement. **Supporting Fig. S4:** TEM micrographs of negatively stained PNIPAm‐based microgels containing 5 mol% of the respective comonomer (MAAc, AAm, or DMAEMA). **Supporting Fig. S5:** Representative SLS measurements of microgel dispersions at 5 mol% comonomer content at the indicated concentrations. (Top) Scattering profiles of anionic microgel dispersions: an initial body‐centered cubic arrangement is obtained at low concentrations, based on relative peak positions of the structure factor *S*(*q*). (Bottom) Corresponding scattering profiles for neutral and cationic microgel dispersions obtained at low concentrations. The scattering profile for neutral microgels shows a similar structure factor trend as for the anionic microgels, while that of the cationic microgels indicates packing without long‐range order. **Supporting Fig. S6:** (a) DLS‐derived hydrodynamic diameter of PNIPAm‐based microgels containing MAAc after 1‐h equilibration in NaCl solutions of varying concentration. Diameters were normalized to the value measured in pure water for each sample. (b) Peak reflectance wavelength shifts (Δ*λ*_max_) of structurally colored assemblies (40 mg mL^−1^) upon NaCl addition. Shifts are reported relative to their respective control aliquot supplemented with water. **Supporting Fig. S7:** Time‐dependent reflectometry of colloidal crystals (40 mg mL^−1^ microgels) prepared from neutral microgels containing 0 mol% AAm (left) and 10 mol% AAm (right). Heating to above the VPTT was initiated after 30 s (dashed arrow). **Supporting Table S1:** Summary of synthesized anionic p(NIPAm‐*co‐*MAAc) microgel library with monomer ratio variation. SDS concentration is fixed at 2.3 mM. Indicated are the used MAAc content, the hydrodynamic diameter *D*_h_ (*Z*‐average), the PDI, and the *ζ*‐potential (all given as an average of three measurements). The volume phase transition temperature (VPTT) was determined as the sigmoidal fit inflection point of the temperature‐response curves. An approximate yield of the reaction was determined by weighing a freeze‐dried aliquot of the product suspension. **Supporting** **Table S2:** Summary of synthesized anionic p(NIPAm‐*co‐*MAAc) microgel library with SDS concentration variation. MAAc content is fixed at 5% of total monomer. Indicated are the used SDS concentrations, the hydrodynamic diameter *D*_h_ (*Z*‐average), the PDI, and the *ζ*‐potential (all given as an average of three measurements). The VPTT was determined as the sigmoidal fit inflection point of the temperature‐response curves. An approximate yield of the reaction was determined by weighing a freeze‐dried aliquot of the product suspension. **Supporting** **Table S3:** Summary of synthesized neutral p(NIPAm‐*co‐*AAm) microgel library with monomer ratio variation. SDS concentration is fixed at 0.9 mM. Indicated are the used AAm content, the hydrodynamic diameter *D*_h_ (*Z*‐average), the PDI, and the *ζ*‐potential (all given as an average of three measurements). The volume phase transition temperature (VPTT) was determined as the sigmoidal fit inflection point of the temperature‐response curves. An approximate yield of the reaction was determined by weighing a freeze‐dried aliquot of the product suspension. **Supporting** **Table S4:** Summary of synthesized neutral p(NIPAm‐*co‐*AAm) microgel library with SDS concentration variation. AAm content is fixed at 5% of total monomer. Indicated are the used SDS concentrations, the hydrodynamic diameter *D*_h_ (*Z*‐average), the PDI, and the *ζ*‐potential (all given as an average of three measurements). The volume phase transition temperature (VPTT) was determined as the sigmoidal fit inflection point of the temperature‐response curves. An approximate yield of the reaction was determined by weighing a freeze‐dried aliquot of the product suspension. **Supporting** **Table S5:** Summary of synthesized cationic p(NIPAm‐*co‐*DMAEMA) microgel library with monomer ratio variation. No surfactant was used in the synthesis. Indicated are the used DMAEMA content, the hydrodynamic diameter *D*_h_ (*Z*‐average), the PDI, and the *ζ*‐potential (all given as an average of three measurements). The VPTT was not determined due to a undefined phase transition. An approximate yield of the reaction was determined by weighing a freeze‐dried aliquot of the product suspension. **Supporting** **Table S6:** Summary of synthesized cationic p(NIPAm‐*co‐*DMAEMA) microgel library with DTAB concentration variation. DMAEMA content is fixed at 5% of total monomer. Indicated are the used DTAB concentrations, the hydrodynamic diameter *D*_h_ (*Z*‐average), the PDI, and the *ζ*‐potential (all given as an average of three measurements). The VPTT was not determined due to a undefined phase transition. An approximate yield of the reaction was determined by weighing a freeze‐dried aliquot of the product suspension. **Supporting** **Table S7:** Summary of the reflectance peak parameters extracted from Figure 8a for assemblies prepared from anionic, neutral, and cationic microgels (5 mol% comonomer) at different microgel concentrations (non‐normalized spectra). Peak wavelength and intensity correspond to the wavelength and magnitude of the first‐order reflection band; FHNW corresponds to the full‐width at half‐maximum. **Supporting** **Table S8:** pH values of microgel assemblies (initial concentration 40 mg mL^−1^) measured after acid/base addition. Samples were diluted to half concentration with water prior to measurement to allow the in situ pH determination under conditions closely approximating the initial experimental system.

## Author Contributions

Conceptualization: Cornelia G. Palivan. Methodology: Manuel Kraus and Mirela Malekovic. Investigation and visualization: Manuel Kraus. Funding acquisition: Cornelia G. Palivan. Writing – original draft: Manuel Kraus, Mirela Malekovic, and Ionel Adrian Dinu. Writing – review and editing: all authors.

## Funding

This study was supported by Universität Basel and Swiss Nanoscience Institute.

## Conflicts of Interest

The authors declare no conflicts of interest.

## Supporting information

Supplementary Material

## Data Availability

The data that support the findings of this study are available from the corresponding author upon reasonable request.
