# Supplementary material for: Tunable Structural Color in Copolymer Microgels Through Controlled Synthesis and Thermally Induced Assembly
Source: Small Sci. 2026 Mar 30;6(4):e202600001. doi: 10.1002/smsc.202600001 (PMC13154912; doi:10.1002/smsc.202600001)
Supplement: Supplementary file 1 — Supplementary Material [file SMSC-6-e202600001-s001.pdf]

Supporting Information

Tunable Structural Color in Copolymer Microgels through Controlled Synthesis and Thermally Induced Assembly

Manuel Kraus, Mirela Malekovic, Ionel Adrian Dinu, Cornelia G. Palivan**

E-mail: cornelia.palivan@unibas.ch, adrian.dinu@unibas.ch

Anionic p(NIPAm-*co*-MAAc) microgel library details

Table S1. Summary of synthesized anionic p(NIPAm-*co*-MAAc) microgel library with monomer ratio variation. SDS concentration is fixed at 2.3 mM. Indicated are the used MAAc content, the hydrodynamic diameter D_h (Z-Average), the PDI, and the ζ-potential (all given as an average of three measurements). The volume phase transition temperature (VPTT) was determined as the sigmoidal fit inflection point of the temperature-response curves. An approximate yield of the reaction was determined by weighing a freeze-dried aliquot of the product suspension.

MAAc content [mol.%]	D_h [nm]	PDI	ζ-potential [mV]	VPTT (°C)	Reaction Yield [%]
0	185 ± 1	0.05	-13.9 ± 0.4	32.2	~24
5	304 ± 7	0.02	-29.6 ± 2.0	33.3	~56
10	372 ± 1	0.02	-42.3 ± 0.5	32.8	~83
15	412 ± 4	0.05	-42.4 ± 0.6	32.2	~78
20	356 ± 3	0.02	-39.9 ± 0.7	33.1	~96
25	331 ± 2	0.02	-46.2 ± 0.9	31.3	~98

Table S2. Summary of synthesized anionic p(NIPAm-*co*-MAAc) microgel library with SDS concentration variation. MAAc content is fixed at 5% of total monomer. Indicated are the used SDS concentrations, the hydrodynamic diameter D_h (Z-Average), the PDI, and the ζ-potential (all given as an average of three measurements). The VPTT was determined as the sigmoidal fit inflection point of the temperature-response curves. An approximate yield of the reaction was determined by weighing a freeze-dried aliquot of the product suspension.

**SDS [mM]**	**D_h [nm]**	**PDI**	**ζ-potential [mV]**	**VPTT (°C)**	**Reaction Yield [%]**
0	672 ± 9	0.07	-27.1 ± 1.2	33.5	~75
0.35	565 ± 3	0.04	-34.3 ± 0.6	33.0	~75
0.69	478 ± 4	0.03	-29.5 ± 2.2	32.7	~62
1.39	413 ± 4	0.02	-27.2 ± 1.8	33.3	~66
2.29	304 ± 7	0.02	-29.6 ± 2.0	33.3	~56
3.47	275 ± 4	0.04	-33.6 ± 1.0	34.0	~54

Neutral p(NIPAm-*co*-AAm) microgel library details

Table S3. Summary of synthesized neutral p(NIPAm-*co*-AAm) microgel library with monomer ratio variation. SDS concentration is fixed at 0.9 mM. Indicated are the used AAm content, the hydrodynamic diameter D_h (Z-Average), the PDI, and the ζ-potential (all given as an average of three measurements). The volume phase transition temperature (VPTT) was determined as the sigmoidal fit inflection point of the temperature-response curves. An approximate yield of the reaction was determined by weighing a freeze-dried aliquot of the product suspension.

AAm content [mol.%]	D_h [nm]	PDI	ζ-potential [mV]	VPTT (°C)	Reaction Yield [%]
0	293 ± 4	0.11	-16.5 ± 0.6	32.2	~83
5	298 ± 2	0.03	-19.8 ± 0.7	34.3	~82
10	332 ± 3	0.05	-12.4 ± 0.6	36.2	~58
15	365 ± 6	0.03	-16.6 ± 0.4	38.4	~48
20	422 ± 2	0.1	-18.4 ± 0.8	41.8	~36

Table S4. Summary of synthesized neutral p(NIPAm-*co*-AAm) microgel library with SDS concentration variation. AAm content is fixed at 5% of total monomer. Indicated are the used SDS concentrations, the hydrodynamic diameter D_h (Z-Average), the PDI, and the ζ-potential (all given as an average of three measurements). The volume phase transition temperature (VPTT) was determined as the sigmoidal fit inflection point of the temperature-response curves. An approximate yield of the reaction was determined by weighing a freeze-dried aliquot of the product suspension.

SDS [mM]	D_h [nm]	PDI	ζ-potential [mV]	VPTT (°C)	Reaction Yield [%]
0	650 ± 6	0.07	-16.1 ± 0.2	33.4	~74
0.69	387 ± 7	0.03	-25.9 ± 0.2	34.4	~76
0.86	298 ± 2	0.03	-19.8 ± 0.7	34.3	~82
1.04	285 ± 3	0.03	-15.8 ± 0.6	34.4	~35
1.21	271 ± 2	0.05	-17.4 ± 0.6	34.4	~44
1.39	240 ± 3	0.05	-8.75 ± 0.3	34.2	~17

Cationic p(NIPAm-*co*-DMAEMA) microgel library details

Table S5. Summary of synthesized cationic p(NIPAm-*co*-DMAEMA) microgel library with monomer ratio variation. No surfactant was used in the synthesis. Indicated are the used DMAEMA content, the hydrodynamic diameter D_h (Z-Average), the PDI, and the ζ-potential (all given as an average of three measurements). The VPTT was not determined due to a undefined phase transition. An approximate yield of the reaction was determined by weighing a freeze-dried aliquot of the product suspension.

DMAEMA content [mol.%]	D_h [nm]	PDI	ζ-potential [mV]	VPTT (°C)	Reaction Yield [%]
0	645 ± 7	0.07	-24.1 ± 0.2	31.5	~87
5	510 ± 3	0.05	14.1 ± 0.1	39.4	~71
10	261 ± 5	0.16	15.4 ± 0.7	39.7	<10
15	238 ± 3	0.17	19.5 ± 0.7	40.2	<10

Table S6. Summary of synthesized cationic p(NIPAm-*co*-DMAEMA) microgel library with DTAB concentration variation. DMAEMA content is fixed at 5% of total monomer. Indicated are the used DTAB concentrations, the hydrodynamic diameter D_h (Z-Average), the PDI, and the ζ-potential (all given as an average of three measurements). The VPTT was not determined due to a undefined phase transition. An approximate yield of the reaction was determined by weighing a freeze-dried aliquot of the product suspension.

DTAB [mM]	D_h [nm]	PDI	ζ-potential [mV]	VPTT (°C)	Reaction Yield [%]
0	510 ± 3	0.05	14.1 ± 0.1	39.4	~71
0.33	490 ± 8	0.11	14.2 ± 0.4	39.8	~68
1.98	441 ± 3	0.03	15.8 ± 0.1	38.7	~68
3.25	391 ± 3	0.03	15.5 ± 1.0	40.0	~65
4.55	349 ± 1	0.05	21.2 ± 0.7	41.4	~42
5.84	308 ± 3	0.02	26.0 ± 0.6	40.9	~39

ATR-FTIR measurements of microgels with varying comonomer content

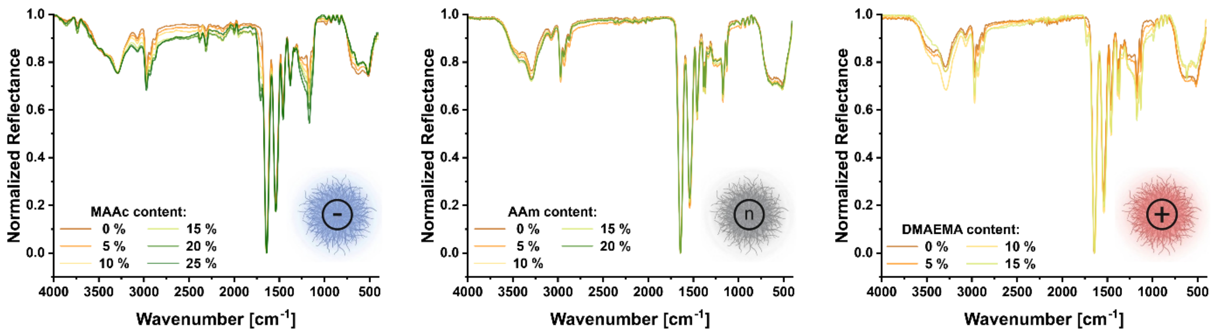

Figure S1. ATR-FTIR spectra of microgels containing varying comonomer contents, normalized to the amide I (C=O) stretching band at 1640 cm^{-1}.

Temperature response upon heating and cooling (hysteresis)

The temperature-dependent swelling/deswelling behavior of the microgels during heating and cooling was evaluated by DLS (temperature range: 15-55 °C, increments of 1°C, 600 s equilibration time per step) (Figure S2). The cooling series was recorded immediately after the heating series to maintain continuous experimental conditions. The hydrodynamic diameters were normalized to the fully swollen value (15 °C) to ensure direct comparison between samples. No measurable hysteresis was observed for the anionic and neutral microgels. The cationic (DMAEMA-containing) microgels showed a slightly delayed re-swelling upon cooling, consistent with their broader and attenuated thermal transition. It is important to note that the fully swollen and fully collapsed endpoints were identical for all types of microgels, indicating a reversible behavior over the investigated temperature range.

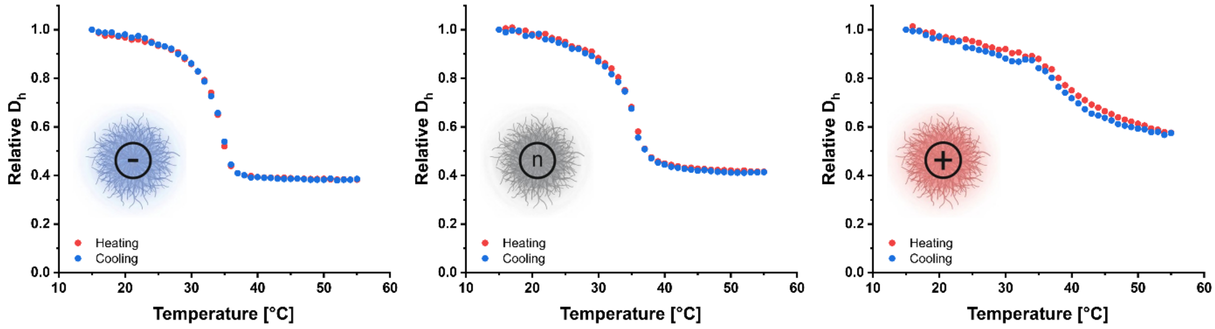

Figure S2. Normalized DLS temperature-induced size response profiles of PNIPAm-based microgels containing 5 mol.% comonomer (left: MAAc; middle: AAm; right: DMAEMA), recorded during consecutive heating (red dots) and cooling (blue dots) from 15 to 55 °C.

Scanning electron microscopy of microgel assemblies

Assemblies of anionic microgels containing 5 mol.% MAAc comonomer at a concentration of 80 mg mL^{-1} were investigated. Scanning electron microscopy (SEM) measurements were performed on a GeminiSEM 450 (Zeiss) operated at an accelerating voltage of 5 kV in secondary-electron mode under high vacuum conditions at room temperature. Samples were sputtered with 20 nm gold before imaging (EM Ace 600, Leica). Cryo-SEM measurements were performed on a XL30 ESEM (Philips) operated at an accelerating voltage of 5 kV under high vacuum conditions and at a temperature of -150°C.

For cryo-SEM, a sample droplet was deposited on a holder and plunge-frozen in nitrogen slush. The sample was then transferred under vacuum into the preparation chamber of the cryo system (Gatan Alto 2500), where it was freeze-fractured and coated with 30 nm of gold. Due to the completely hydrated state of the microgels and low contrast, no distinct particles were observed (Figure S3, left panel). We concluded that imaging would only be possible in the collapsed state of the microgels.

For SEM, two routes were investigated. Firstly, microgel assemblies were frozen in liquid nitrogen and freeze-dried before imaging. However, the lyophilization process created a porous morphology, destroying the assembly (Figure S3, middle panel). Secondly, microgel assemblies were created in a cross-linkable secondary matrix (15% acrylamide monomer, 1 mol.% bis-acrylamide and 1 mol.% 2-hydroxy-2-methylpropiophenone in relation to monomer content) and dried by ethanol solvent switch before imaging. While regular and close-packed arrangements of particles were observed, they represent the dry/collapsed state and do not correspond to the fully swollen assemblies with structural colors (Figure S3, right panel). Therefore, SEM micrographs serve only as qualitative confirmation of the dense microgel arrangements but do not allow the quantitative determination of interparticle distances for the swollen microgel assemblies.

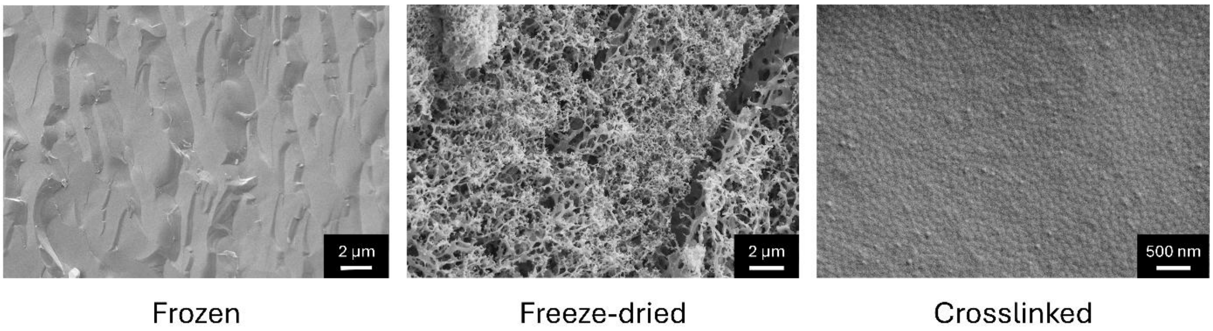

Figure S3. Electron microscopy micrographs of anionic microgel assemblies (5 mol.% MAAc, D_h = 304 nm) in the indicated state. (Left) Cryo-SEM micrograph of a flash-frozen microgel assembly. (Middle) SEM micrograph of a lyophilized microgel assembly. (Right). SEM micrograph of a microgel assembly fixed and dried in a cross-linked secondary matrix to preserve the particle arrangement.

Summary of reflectance peak parameters as a function of microgel type and concentration

Table S7. Summary of the reflectance peak parameters extracted from Figure 8a for assemblies prepared from anionic, neutral, and cationic microgels (5 mol.% comonomer) at different microgel concentrations (non-normalized spectra). Peak wavelength and intensity correspond to the wavelength and magnitude of the first-order reflection band; FHNW corresponds to the full-width at half-maximum.

Comonomer: 5 mol.% MAAc; **Diameter:** 304 nm; **ζ-potential:** -29.6 mV

Microgel Concentration [mg mL^{-1}]	30	40	50	60	70	80
**Peak wavelength [nm]**	669	590	551	513	493	473
**Peak intensity [%]**	41.7	42.0	50.1	68.5	66.3	52.3
**FWHM [nm]**	9	9	5	5	4	5

Comonomer: 5 mol.% AAm; **Diameter:** 387 nm; **ζ-potential:** -25.9 mV

Microgel Concentration [mg mL^{-1}]	30	40	50	60	70	80
**Peak wavelength [nm]**	765	732	670	625	596	580
**Peak intensity [%]**	35.4	32.5	46.3	20.3	28.5	22.4
**FWHM [nm]**	7	11	9	18	9	8

Comonomer: 5 mol.% DMAEMA; **Diameter:** 308 nm; **ζ-potential:** +26.0 mV

Microgel Concentration [mg mL^{-1}]	30	40	50	60
**Peak wavelength [nm]**	630	549	483	459
**Peak intensity [%]**	6.5	6.5	8.4	8.3
**FWHM [nm]**	115	71	47	62

Transmission Electron Microscopy of microgel particles

Transmission electron microscopy (TEM) micrographs of microgel particles were acquired using a CM100 microscope (Philips) operated at an accelerating voltage of 80 kV under vacuum (Figure S4). Microgels containing 5 mol.% comonomer were dispersed in water at concentrations of 0.1 mg mL^{-1}. Aliquots of 5 µl were deposited onto glow-discharged copper grids (400-mesh square) and negatively stained with 2 wt.% phosphotungstic acid (PTA, pH 7) prior to imaging.

The TEM micrographs are consistent with a core–corona (core–shell-like) microgel architecture and show clear morphological differences between cationic and anionic/neutral samples. While the anionic and neutral microgels appear predominantly spherical with comparatively uniform particle shapes, the cationic (DMAEMA-containing) microgels show less-regular morphologies, higher size dispersity, and a strong tendency toward particle–particle association. Although TEM sample preparation involves drying and staining and may accentuate aggregation, these observations support the presence of distinct colloidal behavior in the cationic series.

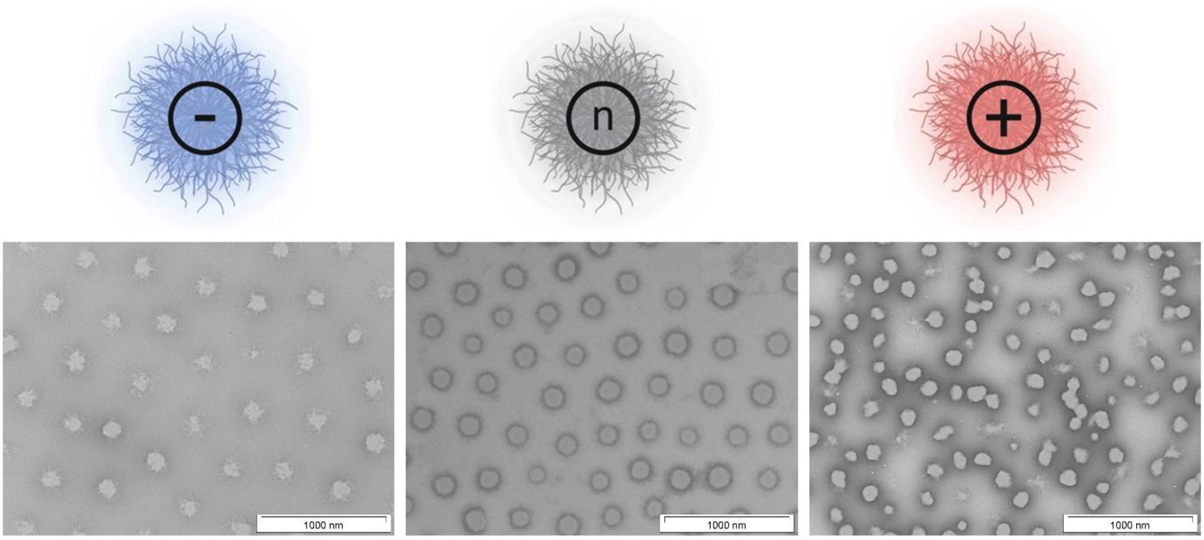

Figure S4. TEM micrographs of negatively stained PNIPAm-based microgels containing 5 mol.% of the respective comonomer (MAAc, AAm, or DMAEMA).

Static Light Scattering characterization of microgel assemblies

Angle-resolved static light scattering (SLS) measurements were performed to evaluate the structural organization of microgel dispersions. The experiments were performed at 20 °C using a multi-angle light scattering spectrometer (LS Instruments, Switzerland) equipped with a 21 mW He–Ne laser (λ_0 = 632.8 nm). Scattering intensities were recorded over an angular range (θ) of 20°–150° with increments of 5°. The scattering vector, q, was calculated according to:[1,2]

$$q = \frac{4\pi n}{\lambda_0} \sin\left(\frac{\theta}{2}\right)$$

where n = 1.33058 is the refractive index of the medium (water).

The measured scattering profiles were corrected for background contributions by subtracting the scattering intensities of water, recorded under identical instrumental conditions. The corrected intensity $I_{corr}(q)$ was normalized to the incident laser intensity and used to calculate the apparent structure factor $S(q)$ according to:

$$S(q) = \frac{I_{corr}(q)}{I_{dil}(q)}$$

Where $I_{dil}(q)$ corresponds to the scattering intensity of the same microgel at very low concentrations used for DLS measurements, where the interparticle correlations are negligible. This procedure allows for the separation of form and structure factor contributions and enables the estimation of interparticle correlations in microgel assemblies.

At concentrations of approximately 4 mg mL^{-1}, anionic and neutral microgel dispersions showed a single, first-order Bragg peak at q values of about 0.015 nm^{-1}. The peak intensity and sharpness depended on the comonomer type, reflecting differences in electrostatic and steric interactions governing the interparticle ordering. Cationic microgel dispersions show evidence of packing without long-range ordering, consistent with an amorphous arrangement.

For the anionic microgels at a concentration of 8 mg mL^{-1}, the primary peak was shifted to 0.018 nm^{-1}, consistent with a decrease in lattice spacing due to the increased particle number density. In addition, two higher-order reflections appeared at 0.021 nm^{-1} and 0.024 nm^{-1}. The relative positions of these peaks highlight the body-centered cubic (BCC) lattice and support the formation of soft microgel crystals with long-range ordering.[3,4] However, at concentrations relevant for structural color formation (20-80 mg mL^{-1}, not depicted), the pronounced turbidity and multiple scattering effects limited a reliable analysis of the peaks, in agreement with previous reports on highly concentrated colloidal dispersions.[4]

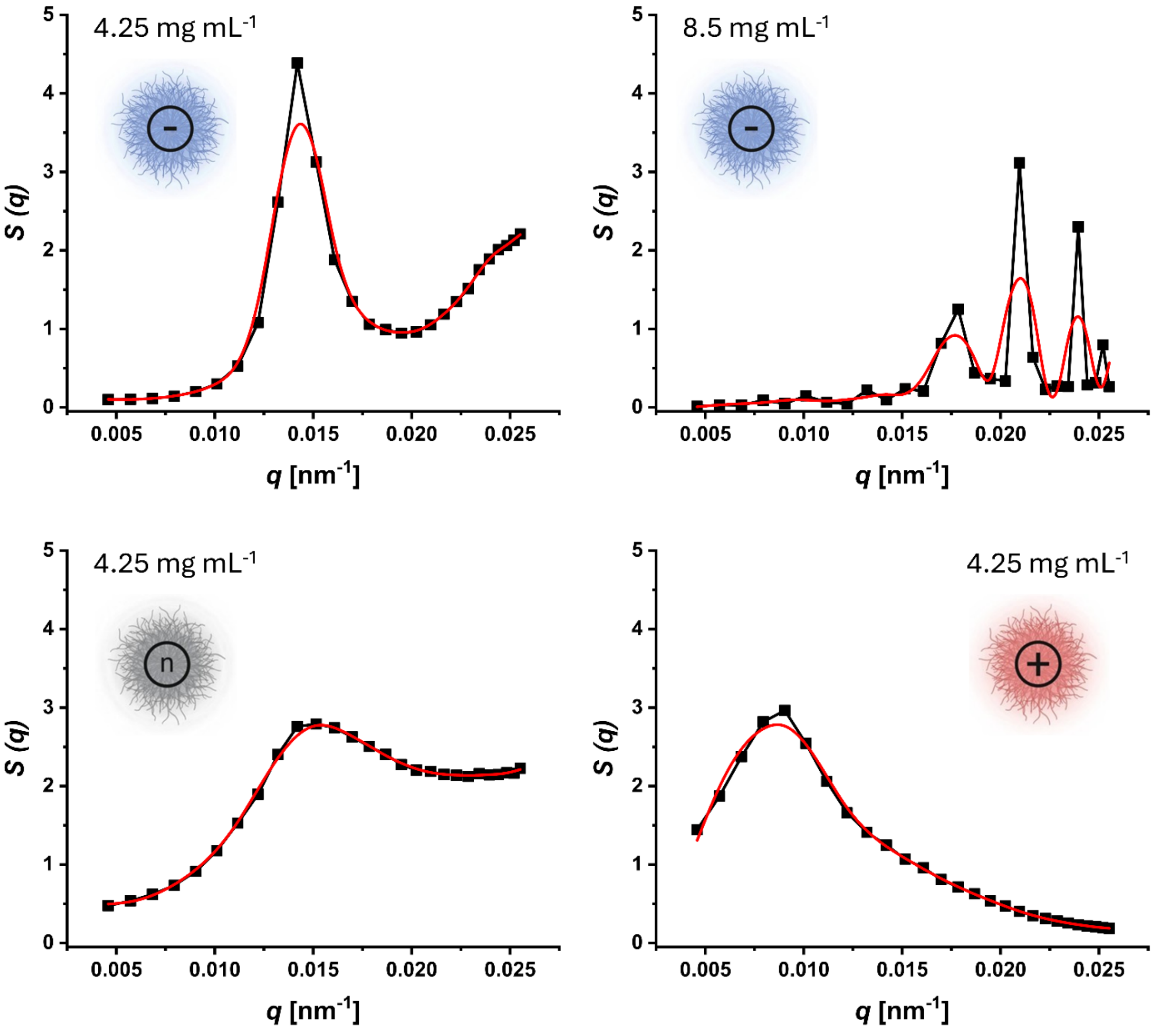

Figure S5. Representative SLS measurements of microgel dispersions at 5 mol.% comonomer content at the indicated concentrations. (Top) Scattering profiles of anionic microgel

dispersions: an initial body-centered cubic arrangement is obtained at low concentrations, based on relative peak positions of the structure factor $S(q)$. (Bottom) Corresponding scattering profiles for neutral and cationic microgel dispersions obtained at low concentrations. The scattering profile for neutral microgels shows a similar structure factor trend as for the anionic microgels, while that of the cationic microgels indicates packing without long-range order.

Determination of pH changes in concentrated microgel assemblies

Selected anionic microgel assemblies (0, 5, and 25 mol.% MAAc comonomer content, microgel concentration: 40 mg mL^{-1}) for pH change measurements were investigated to evaluate the pH changes induced upon addition of acid or base. Direct pH measurements under reflectometry conditions were not feasible due to the small sample volume and the highly viscous character of the dispersions at these concentrations. Therefore, immediately after acid/base addition, the samples were diluted to a total volume of 110 µL with water. This dilution allowed for reliable *in situ* measurements using a pH-meter electrode (InLab Ultra-Micro-ISM, Mettler Toledo) while keeping experimental conditions that closely approximate the chemical environment of the initial concentrated samples.

The measured values show clear and systematic pH trends that agree with the increasing amount of MAAc functional groups. Specifically, the microgel assemblies containing MAAc exhibit a pronounced buffering effect upon the addition of the base, reflecting the protonation/deprotonation equilibrium of the carboxylic acid groups. In contrast, the microgels without MAAc show no comparable pH compensation effect.

It is important to note that an exact determination of the absolute pH within the structurally colored, highly concentrated samples is experimentally inaccessible due to viscosity constraints and electrode limitations. However, the comparative measurements presented here highlight the expected compositional dependence and confirm that the pH trends correlate with both microgel formulation and the observed peak wavelength shifts. Thus, while the absolute pH values cannot be directly quantified in the undiluted microgel assemblies, the obtained results clearly validate the intended pH change and its dependence on MAAc content.

Table S8. pH values of microgel assemblies (initial concentration 40 mg mL^{-1}) measured after acid/base addition. Samples were diluted to half concentration with water prior to measurement to allow the *in situ* pH determination under conditions closely approximating the initial experimental system.

MAAc content [mol.%]	1 M HCl	0.1 mM NaOH	10 mM NaOH	1 M NaOH
0	1.4	4.6	9.7	12.4
5	1.4	4.2	4.9	12.2
25	1.3	4.8	5.4	11.8

Sensitivity of anionic microgels to ionic strength

To assess the sensitivity of anionic microgels to ionic strength, DLS measurements were performed on dilute dispersions of MAAc-containing microgels (0.1 mg mL^{-1}) after equilibration for one hour in aqueous NaCl solutions of various concentrations (Figure S6a). Hydrodynamic diameters were normalized to the corresponding value measured in pure water (not shown on the plot because of the logarithmic scale). In addition, the effect of ionic strength changes on the optical response of concentrated microgel assemblies was evaluated by reflectometry measurements (Figure S6b). Assemblies were prepared using MAAc-containing microgels at a concentration of 40 mg mL^{-1}, and the initial peak reflectance wavelength (λ_max) was determined by reflectometry. Aliquots were then supplemented with 5 µl of NaCl solution at the indicated concentration and re-annealed to allow structural re-equilibration prior to re-measurement. The resulting peak wavelength shifts were referenced to a control aliquot supplemented with water to correct for dilution effects and isolate the salt-induced contribution.

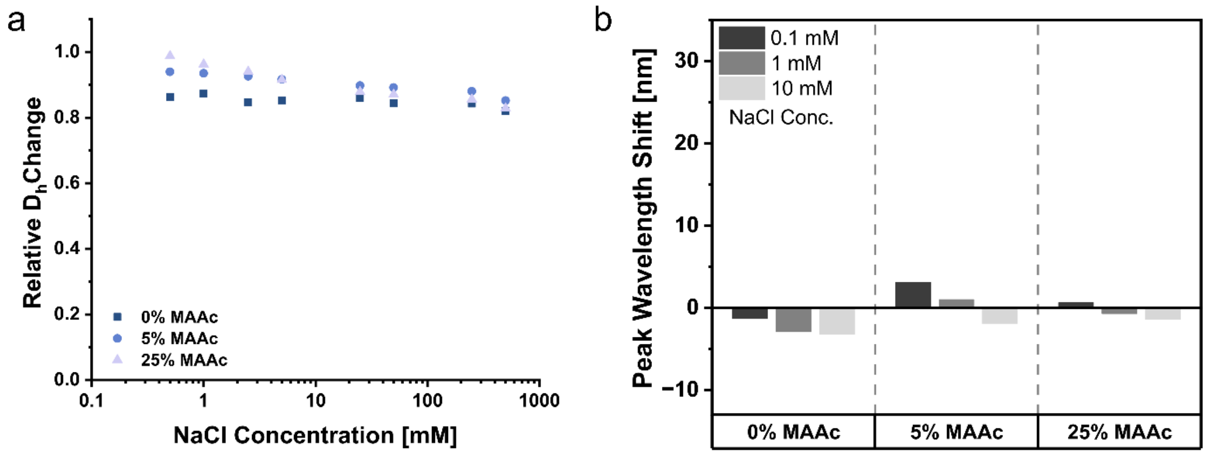

Figure S6. a) DLS-derived hydrodynamic diameter of PNIPAm-based microgels containing MAAc after 1 h equilibration in NaCl solutions of varying concentration. Diameters were normalized to the value measured in pure water for each sample. b) Peak reflectance wavelength shifts ($\Delta\lambda_{max}$) of structurally colored assemblies (40 mg mL^{-1}) upon NaCl addition. Shifts are reported relative to their respective control aliquot supplemented with water.

Time-resolved temperature response of microgel assemblies

To assess the timescale of the optical temperature response, we performed time-resolved reflectometry on neutral AAm-containing microgel assemblies (Figure S7). Assemblies prepared at 40 mg mL^{-1} from microgels containing 0 and 10 mol.% AAm were monitored during a rapid temperature increase to above their respective VPTT. Reflectance spectra were recorded at intervals of 1 s. Samples were first kept at room temperature for 30 s, after which heating was initiated. The reflectance peak decayed rapidly upon heating, reaching a collapsed, low-reflectivity state after ~ 15 s for 0 mol.% AAm and ~ 45 s for 10 mol.% AAm, consistent with the higher VPTT and delayed collapse of the AAm-rich microgels.

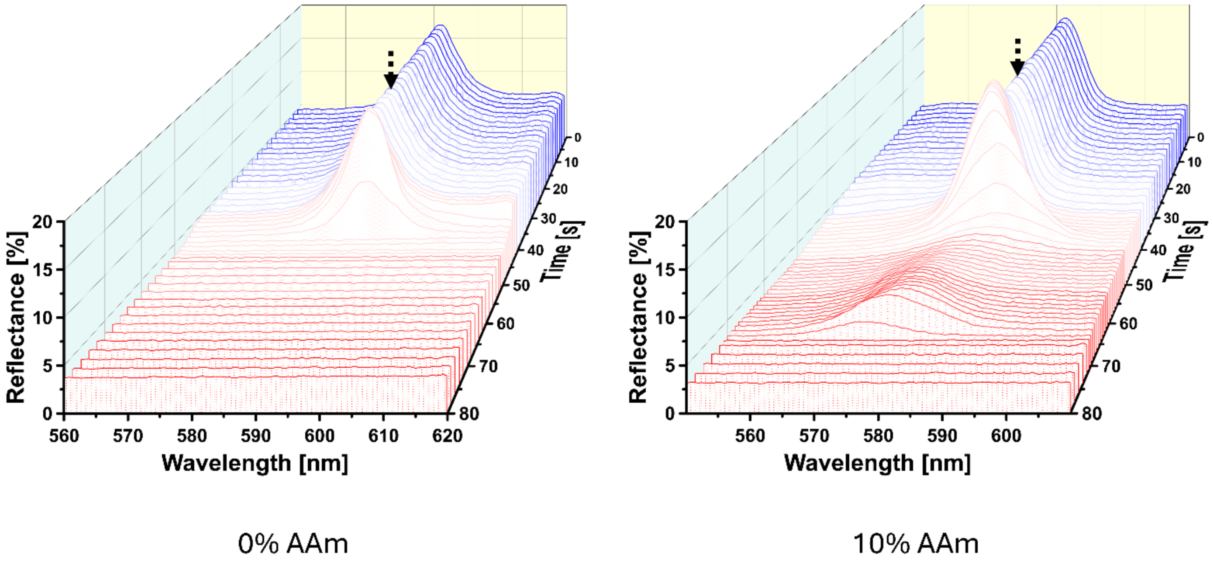

Figure S7. Time-dependent reflectometry of colloidal crystals (40 mg mL^{-1} microgels) prepared from neutral microgels containing 0 mol.% AAm (left) and 10 mol.% AAm (right). Heating to above the VPTT was initiated after 30 s (dashed arrow).

References

[1] R. Klein, G. Nägele, "Static and Dynamic Scattering by Concentrated Systems: Theory," *Current Opinion in Colloid & Interface Science*, **1996** *1* (1) 4, https://doi.org/10.1016/S1359-0294(96)80037-9.

[2] R. Piazza, "Settled and Unsettled Issues in Particle Settling," *Reports on Progress in Physics*, **2014** *77* (5) 056602, https://doi.org/10.1088/0034-4885/77/5/056602.

[3] L. A. Lyon, A. Fernandez-Nieves, "The Polymer/Colloid Duality of Microgel Suspensions," *Annual Review of Physical Chemistry*, **2012** *63* (Volume 63, 2012) 25, https://doi.org/10.1146/annurev-physchem-032511-143735.

[4] P. S. Mohanty, S. Nöjd, K. van Gruijthuijsen, J. J. Crassous, M. Obiols-Rabasa, R. Schweins, A. Stradner, P. Schurtenberger, "Interpenetration of Polymeric Microgels at Ultrahigh Densities," *Scientific Reports*, **2017** *7* (1) 1487, https://doi.org/10.1038/s41598-017-01471-3.
